# The Indo-European Cognate Relationships dataset

**DOI:** 10.1038/s41597-025-05445-3

**Published:** 2025-09-02

**Authors:** Cormac Anderson, Matthew Scarborough, Lechosław Jocz, Martin Joachim Kümmel, Thomas Jügel, Britta Irslinger, Roland Pooth, Henrik Liljegren, Richard F. Strand, Geoffrey Haig, Ulrich Geupel, Martin Macak, Ronald I. Kim, Erik Anonby, Tijmen Pronk, Oleg Belyaev, Tonya Kim Dewey-Findell, Matthew Boutilier, Cassandra Freiberg, Robert Tegethoff, Matilde Serangeli, Krzysztof Stroński, Alexander Falileyev, Nikos Liosis, Kim Schulte, Ganesh Kumar Gupta, Raheleh Izadifar, Patrycja Markus, Nicholas Williams, Simone Loi, Nicholas Sims-Williams, Martin Findell, Shirin Adibifar, Giovanni Abete, Petar Atanasov, Esther Baiwir, Maria-Reina Bastardas, Adam Benkato, Lisa Shugert Bevevino, Éva Buchi, Giorgio Cadorini, Chundra Cathcart, Loïc Cheveau, Charalambos Christodoulou, Jérémie Delorme, Steven N. Dworkin, Deniz Ekici, Shervin Farridnejad, Mojtaba Gheitasi, Harald Hammarström, Steve Hewitt, Afsar Ali Khan, Muhammad Kamal Khan, Liudmila Khokhlova, Deborah Kim, Christopher Lewin, Borana Lushaj, Parvin Mahmoudveysi, Masoud Mahommadirad, Sam Mersch, Baydaa Mustafa, Fatemeh Nemati, Maryam Nourzaei, Peadar Ó Muircheartaigh, Virginia Oogjen, Muhammed Ourang, Heather Pagan, Timothy S. Palmer, Steve Pepper, Mandar Purandare, Khwaja Rehman, Guto Rhys, Unn Røyneland, Muhammad Zaman Sagar, Jade Jørgen Sandstedt, Lars Steensland, Mortaza Taheri-Ardali, Mahnaz Talebi-Dastenaei, Sabine Tittel, Tiago Tresoldi, Michiel de Vaan, Annemarie Verkerk, Arjen Versloot, Paul Videsott, Nikola Vuletić, Manuel Widmer, Arash Zeini, Hans-Jörg Bibiko, Fiona Runge, Russell D. Gray, Paul Heggarty

**Affiliations:** 1 Department of Linguistic and Cultural Evolution, Max Planck Institute for Evolutionary Anthropology, Deutscher Platz 6, 04103 Leipzig, Germany; 2 Surrey Morphology Group, University of Surrey, Guildford, Surrey GU2 7XH UK; 3 Department of Nordic Studies and Linguistics, University of Copenhagen, 2300 København S, Denmark; 4 Faculty of Humanities, Jacob of Paradies University, Fryderyka Chopina 52, 66-400 Gorzów Wielkopolski, Poland; 5 Seminar for Indo-European Studies, Institut für Orientalistik, Indogermanistik, Ur- und Frühgeschichtliche Archäologie, Friedrich-Schiller-Universität Jena, Jena, Germany; 6 Ruhr-Universität Bochum, CERES - Center of Religious Studies, Universitätsstr. 90a, 44789 Bochum, Germany; 7 Saxon Academy of Sciences and Humanities, Karl-Tauchnitz-Straße 1, 04107 Leipzig, Germany; 8 Department of Linguistics, Ghent University, Blandijnberg 2, 9000 Ghent, Belgium; 9 Department of Linguistics, Stockholm University, Universitetsvägen 10 C, Frescati, 10691 Stockholm, Sweden; 10 Independent scholar, Cottonwood, Arizona USA; 11 Department of General Linguistics, University of Bamberg, Schillerplatz 17, 96047 Bamberg, Germany; 12 Vergleichende Sprachwissenschaft und Keltologie, Philipps-Universität, Wilhelm-Röpke-Str. 6E, 35032 Marburg, Germany; 13 Lehrstuhl für Vergleichende Sprachwissenschaft, Julius-Maximilians-Universität Würzburg, Oswald-Külpe-Weg 84, 97074 Würzburg, Germany; 14 Independent scholar, Bratislava, Slovakia; 15 Department of Older Germanic Languages, Faculty of English, Adam Mickiewicz University in Poznań, ul. Grunwaldzka 6, 60-780 Poznań, Poland; 16 School of Linguistics and Language Studies, Carleton University, 1125 Colonel By Drive, Ottawa, Ontario K1S 5B6 Canada; 17 Leiden University Centre for Linguistics, Postbus 9515, 2300 RA Leiden, Netherlands; 18 Institute of Linguistics of the Russian Academy of Sciences, Bolshoi Kislovsky lane 1/1, 125009 Moscow, Russia; 19 Centre for the Study of the Viking Age, School of English, Trent Building, University of Nottingham, Nottingham, UK; 20 Department of German, Nordic and Slavic, University of Wisconsin-Madison, Madison, Wisconsin USA; 21 Independent scholar, Berlin, Germany; 22 Max Planck Institute of Geoanthropology, Kahlaische Strasse 10, 07745 Jena, Germany; 23 Dipartimento di Promozione delle Scienze Umane e della Qualità della Vita, Università di Roma San Raffaele, Rome, Italy; 24 Institute of Oriental Studies, Faculty of Modern Languages, Adam Mickiewicz University in Poznań, Grunwaldzka 6, 61-780 Poznań, Poland; 25 Departamento de Filología Clásica e Indoeuropeo, University of Salamanca, Palacio de Anaya (Filología), Plaza de Anaya s/n, 37008 Salamanca, Spain; 26 Institute of Modern Greek Studies, Aristotle University of Thessaloniki, 54124 Thessaloniki, Greece; 27 Department of Translation and Communication, Jaume I University, 12071 Castellón de la Plana, Castellón Spain; 28 Department of Linguistics, Faculty of Humanities, Bu-Ali Sina University, Shahid Mostafa Ahmadi Roshan Street, Hamedan, 6516738695 Iran; 29 University College Dublin, Newman, Building, Block A, Level 2, Stillorgan Rd, Belfield, Dublin, D04 T6C2 Ireland; 30 Institut für Linguistik, Philologische Fakultät, Universität Leipzig, 04081 Leipzig, Germany; 31 SOAS University of London, Thornhaugh Street, Russell Square, London, WC1H 0XG UK; 32 School of English, University of Nottingham, University Park, Nottingham, NG7 2RD UK; 33 Dipartimento di Studi Umanistici, Università degli Studi di Napoli Federico II, Via Porta di Massa, 1, 80133 Napoli, Italy; 34 Independent scholar, Skopje, North Macedonia; 35 Faculté des Humanités, Université de Lille, Domaine Universitaire du Pont de Bois, 3 Rue du Barreau, 59650 Villeneuve-d’Ascq, France; 36 Departament de Filologia Clàssica, Romànica i Semítica, Facultat de Filologia i Comunicació, Universitat de Barcelona, Gran Via de les Corts Catalanes, 585, 08007 Barcelona, Spain; 37 Middle Eastern Languages and Cultures, UC Berkeley, 250 Social Sciences Building, Berkeley, CA 94720 USA; 38 French and Latin Faculty, University of Minnesota, Morris, Division of Humanities, 600 East Fourth Street, Morris, Minnesota 56267 USA; 39 ATILF (CNRS & Université de Lorraine), 44 avenue de la Libération, B.P. 30687, 54063 Nancy, France; 40 Department of Classical Studies, Philosophical Faculty, Masaryk University, Gorkého 63/14, 602 00 Brno, Czechia; 41 Institute for the Interdisciplinary Study of Language Evolution, University of Zurich, Affolternstrasse 56, 8050 Zürich, Switzerland; 42 Université Rennes 2, Place du recteur Henri Le Moal, CS 24307, 35043 Rennes, France; 43 University of Cyprus, P.O. Box 20537, 1678 Nicosia, Cyprus; 44 LACITO - UMR 7107 - CNRS / Paris III/Inalco, Langues et civilisations à tradition orale, 7 rue Guy Môquet (bât. D), 94801 Villejuif, France; 45 Romance Linguistics, 108 Modern Languages Building, 812 East Washington Street, Ann Arbor, Michigan 48109-1275 USA; 46 Independent scholar, Berkeley, USA; 47 Universität Hamburg, Geschichte und Kultur des Vorderen Orients, Edmund-Siemers-Allee 1, Flügel Ost, 20146 Hamburg, Germany; 48 University of Ilam, Ilam Province, Ilam, Pazhohesh Blvd Iran; 49 Department of Linguistics and Philology, Uppsala University, Box 635, 751 26 Uppsala, Sweden; 50 Independent scholar, Östra Ämtervik, Sweden; 51 Consultant Language Development, FLI 2, Block 19. G-8 Markaz, Islamabad, 44000 Pakistan; 52 Department of English Language and Applied Linguistics, Allama Iqbal Open University, Islamabad, Pakistan; 53 Department of Indian Philology, Institute of Asian and African Studies, Moscow State University, Mokhovaya 11, Moscow, 125009 Russia; 54 Independent scholar, Grand Forks, USA; 55 Faclair na Gàidhlig, Sabhal Mòr Ostaig, Sleat, Isle of Skye IV44 8RQ UK; 56 Radboud University, Houtlaan 4, 6525 XZ Nijmegen, Netherlands; 57 Department of History and Culture of the Middle East, Asia Africa Institute, University of Hamburg, Hamburg, Germany; 58 UMR 8041 Centre de recherche sur le monde iranien, CNRS – Campus Paris-Villejuif, 7 rue Guy Môquet, 94800 Villejuif, France; 59 Faculty of Asian and Middle Eastern Studies, University of Cambridge, Sidgwick Avenue, Cambridge, CB3 9DA UK; 60 Faculty of Language and Literature, Humanities, Arts and Education, Université du Luxembourg, Maison des Sciences Humaines, 11 Porte des Sciences, L-4366 Esch-sur-Alzette, Luxembourg; 61 Department of English Language and Literature, Persian Gulf University, Bushehr, 7516913817 Iran; 62 Celtic and Scottish Studies, College of Arts, Humanities and Social Sciences, University of Edinburgh, 57 George Square, Edinburgh, EH8 9JU UK; 63 University of New South Wales, Sydney, NSW 2052 Australia; 64 School of Humanities, University of Westminster, 309 Regent Street, London, W1B 2HW UK; 65 Department of Linguistics and Scandinavian Studies, University of Oslo, P.O. Box 1102, Blindern, 0317 OSLO Norway; 66 Directorate of Higher Education Colleges, Muzaffarabad, Azad Jammu & Kashmir Pakistan; 67 Independent scholar, Brussels, Belgium; 68 Center for Multilingualism in Society across the Lifespan, University of Oslo, PO box 1102, Blindern, 0317 OSLO Norway; 69 Forum for Language Initiatives, P.O Box No. 763, Islamabad, Pakistan; 70 Volda University College, Postboks 500, 6101 Volda, Norway; 71 UiT - The Arctic University of Norway, Postboks 6050 Langnes, 9037 Tromsø, Norway; 72 Independent scholar, Lund, Sweden; 73 Department of English, Shahrekord University, Shahrekord, Iran; 74 Department of Linguistics, Alzahra University, Vanak, Tehran, Iran; 75 Heidelberg Academy of Sciences and Humanities, Karlstraße 4, 69117 Heidelberg, Germany; 76 Universität Basel, Department of Ancient Civilizations, Historical-Comparative Linguistics, Petersgraben 51, CH-4051 Basel, Switzerland; 77 Department of Language Science and Technology, Saarland University, 66123 Saarbrücken, Germany; 78 Germaanse taalkunde, in het bijzonder de Duitse, Scandinavische en Friese talen, Faculteit der Geesteswetenschappen, Postbus 1641, 1000 BP Amsterdam, Netherlands; 79 Faculty of Education, Free University of Bozen-Bolzano, Regensburger Allee 16, 39042 Brixen, Italy; 80 Center for Adriatic Onomastics and Ethnolinguistics, University of Zadar, Trg kneza Višeslava 9, 23000 Zadar, Croatia; 81 NCCR Evolving Language, Affolternstrasse 56, 8050 Zürich, Switzerland; 82 Faculty of Asian and Middle Eastern Studies, University of Oxford, Pusey Lane, Oxford, OX1 2LE UK; 83 Independent scholar, Leipzig, Germany; 84 School of Psychology, University of Auckland, 23 Symonds St., Auckland, 1010 New Zealand; 85 WAVES group, Dept of Human Behavior, Ecology and Culture, Max Planck Institute for Evolutionary Anthropology, Deutscher Platz 6, 04103 Leipzig, Germany; 86 Language and the Anthropocene Group, Max Planck Institute of Geoanthropology, Kahlaische Strasse 10, 07745 Jena, Germany; 87 Departamento de Humanidades, Pontificia Universidad Católica del Perú, Av. Universitaria 1801, San Miguel, 15088 Lima, Peru

**Keywords:** Social anthropology, Human behaviour

## Abstract

The Indo-European Cognate Relationships (IE-CoR) dataset is an open-access relational dataset showing how related, inherited words (‘cognates’) pattern across 160 languages of the Indo-European family. IE-CoR is intended as a benchmark dataset for computational research into the evolution of the Indo-European languages. It is structured around 170 reference meanings in core lexicon, and contains 25731 lexeme entries, analysed into 4981 cognate sets. Novel, dedicated structures are used to code all known cases of horizontal transfer. All 13 main documented clades of Indo-European, and their main subclades, are well represented. Time calibration data for each language are also included, as are relevant geographical and social metadata. Data collection was performed by an expert consortium of 89 linguists drawing on 355 cited sources. The dataset is extendable to further languages and meanings and follows the Cross-Linguistic Data Format (CLDF) protocols for linguistic data. It is designed to be interoperable with other cross-linguistic datasets and catalogues, and provides a reference framework for similar initiatives for other language families.

## Background & Summary

### Background: the Indo-European languages and phylogenetic research

Almost half of the world’s population speaks a language of the Indo-European lineage^1^. This huge family of over 400 languages has a long research tradition stretching back well over two hundred years, but much remains to be understood about its origins, dispersal, and internal structure. In particular, major phylogenetic analyses in recent years, as surveyed in^2^, have supported conflicting hypotheses for the time depth and geographical origin of Indo-European^3–8^. Recent analyses have mostly used state-of-the-art Bayesian phylogenetic analysis tools, applied to datasets of cognates (related words) across the Indo-European languages, i.e. forerunners of the new IE-CoR dataset presented here. Those past datasets have been criticised^2,8–11^, however, for their limited and uneven coverage of the Indo-European family through time and space, and across its internal diversity, as well as for poor data coding — data problems directly implicated in the inconsistent phylogenetic results obtained^2^.

The new Indo-European Cognate Relationships (IE-CoR) dataset is designed to overcome the limitations of past datasets. It encodes cognate relationships in 170 meanings of core vocabulary (i.e. basic terms like hand, drink, black, three) across 160 Indo-European languages. (For explanations of linguistic terminology used in this text, such as ‘cognate’, see the Definitions box.) IE-CoR aims to provide a benchmark dataset for quantitative and phylogenetic research on the Indo-European (IE) language family.

### Summary of the IE-CoR Dataset

**Fig. 1:**
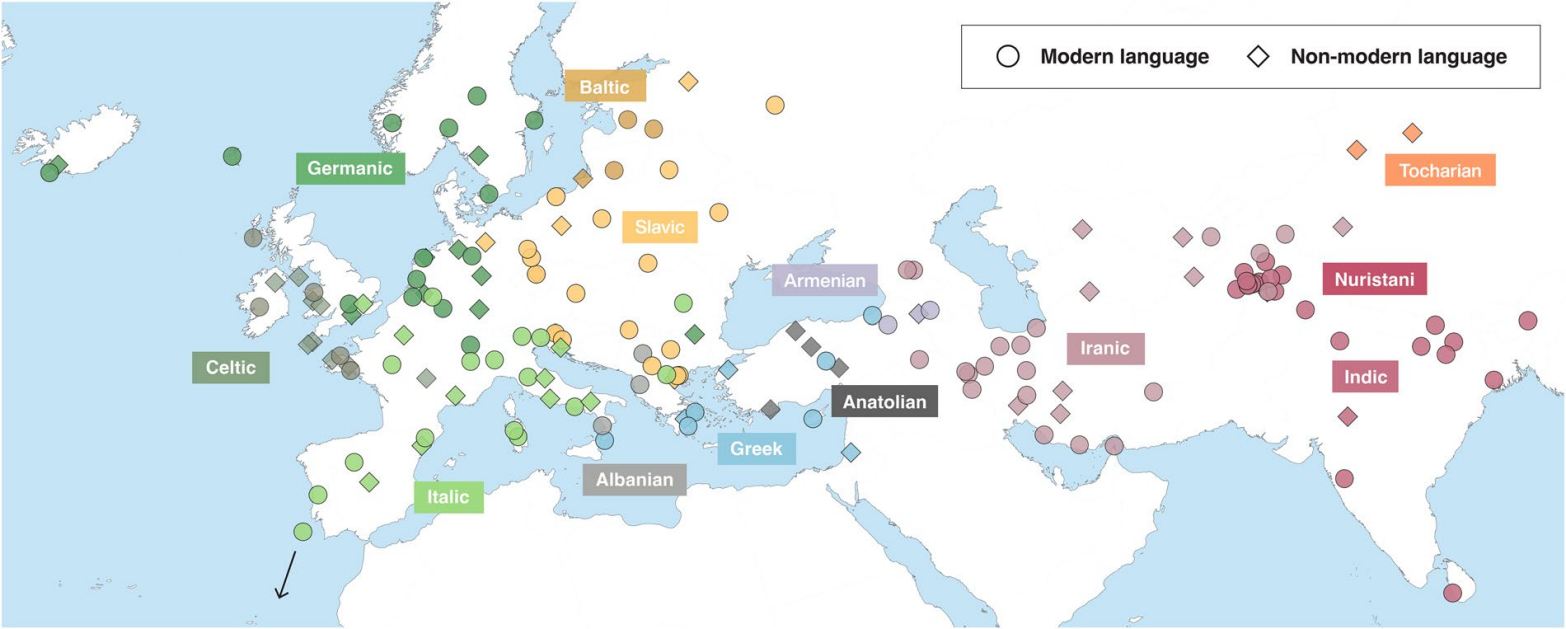
Language sample in IE-CoR 1.2. Colours represent main clades.

In total, the initial data contribution for IE-CoR, version 1.2, covers 160 languages, 52 of them historically attested languages with date calibrations before the present, across all 13 major documented clades of Indo-European (see Fig. 1). All IE-CoR languages are real, documented languages, not undocumented, idealised proto-languages^2,3^. For each of these languages, IE-CoR documents the primary attested lexeme for the same 170 comparison meanings. All 25781 lexemes in the dataset are grouped into one of 4981 cognate sets, with consistent protocols for dealing with morphological complexity and loanwords. Sources for data and encoding decisions are given by 10925 citations to 355 publications.

**Fig. 2:**
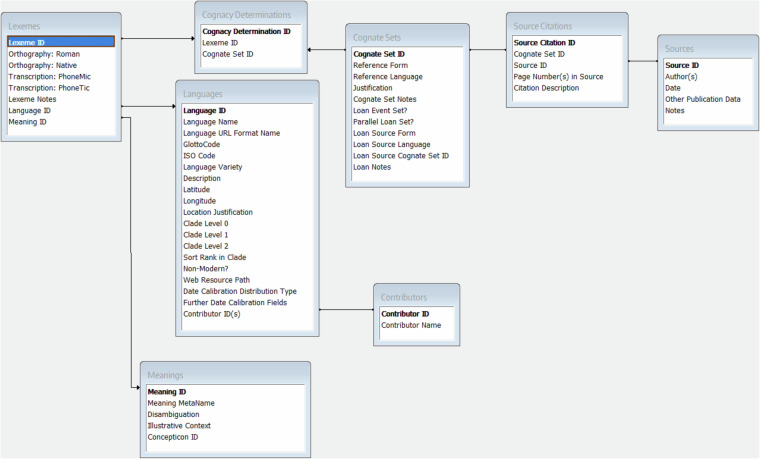
Schematic, simplified overview of the relationships between fields in the main tables of the IE-CoR dataset.

**Fig. 3:**
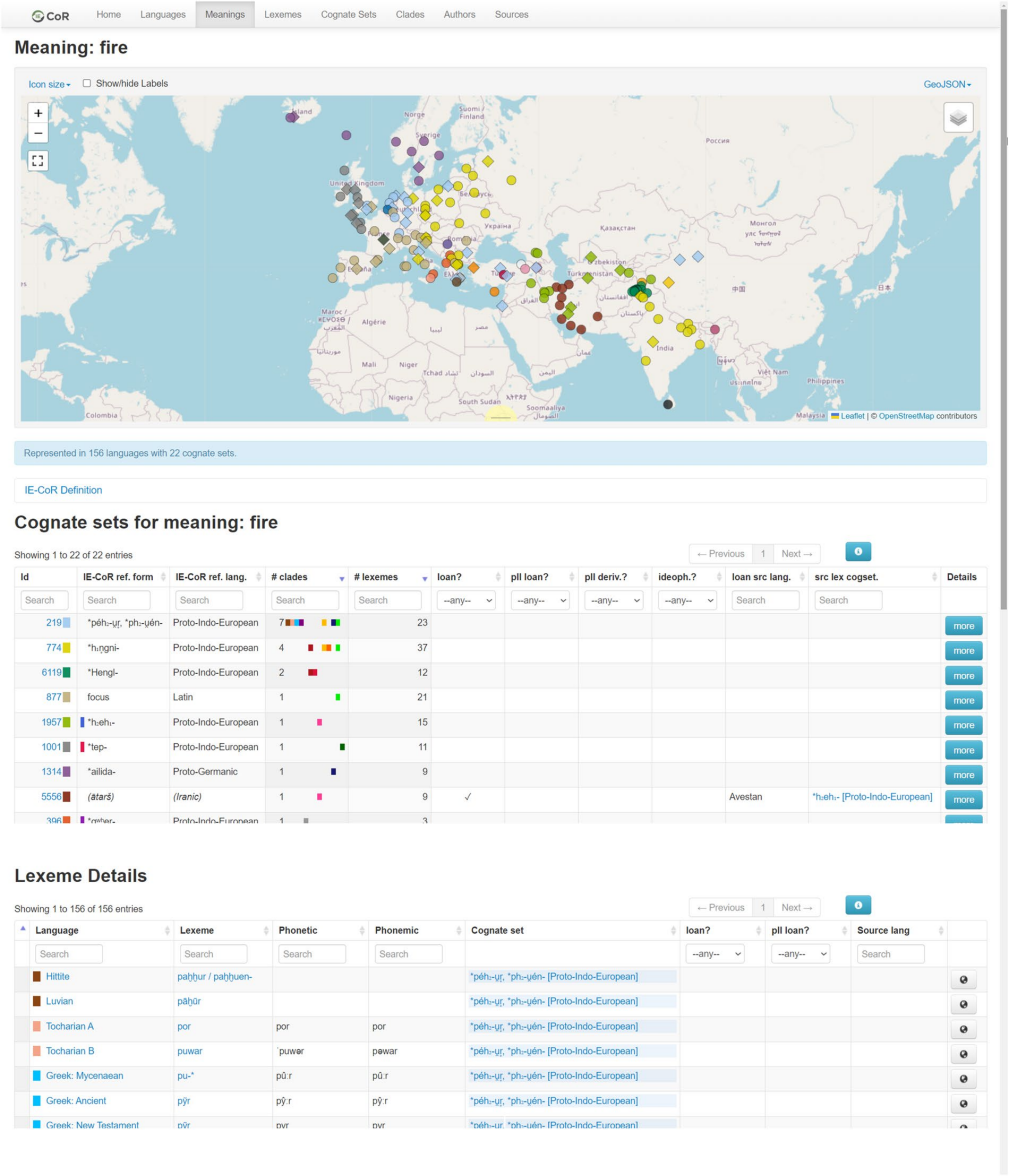
Illustration of cognate sets and lexemes across the Indo-European language family in the IE-CoR dataset, for the example meaning FIRE. An interactive version is available at iecor.clld.org/parameters/fire.

IE-CoR is published under the Cross-Linguistic Linked Data project (CLLD: https://github.com/clld/clld)^12^, which develops and curates interoperable data publication structures using Linked Data principles. It follows the Cross-Linguistic Data Format (CLDF: https://cldf.clld.org)^13,14^, which provides a standard format and guidelines for storing linguistic datasets as interrelated plain text files, facilitating version control, long-term archiving, and FAIR access. As well as the raw data files (see link under ‘Dataset’ below), the IE-CoR dataset can be explored through the dedicated IE-CoR CLLD web application at https://iecor.clld.org. A schematic overview of the dataset structure and relationships is given in Fig. 2. Meanwhile, Fig. 3 illustrates how cognate sets pattern across the IE-CoR language sample for the example meaning fire.

Importantly, the data point in which languages are compared in the IE-CoR dataset is focused by a set of narrow qualifications. The point of comparison is the cognacy state of the *root morpheme* of the *primary* lexeme used in the *IE-CoR target sense* of each reference meaning. For much more detail on how IE-CoR defines and applies these qualifications, see the corresponding subsections within ‘Methods’ below.

Important also is to clarify that IE-CoR is a strictly comparative dataset. Any dataset of a subset of core lexicon can only represent a sample, a fraction of any language’s entire lexicon. So IE-CoR is not, and necessarily did not aspire to be, a dictionary or thesaurus resource. Nor is it a dataset of all known cognate forms across the Indo-European family (reference works of these feature ubiquitously among our citations instead). Rather, IE-CoR focuses only on its particular subset of 170 word meanings, and for each of them its purpose is certainly not to extract as broad a coverage as possible of the full range and lexical wealth of any language in that general semantic field. On the contrary, to ensure the absolute priority of a strictly *consistent dataset* (across all languages covered) [^2^Fig. 3], IE-CoR introduces its own strict, narrow definitions and specifications of each of its 170 reference meanings. In each one, IE-CoR aims to record only the primary lexeme in each language, and its cognate state. Many famous Indo-European cognate sets do not come into IE-CoR, then, if they are not used in any IE-CoR reference meaning. IE-CoR records no cognates of the numerals six to ten, for example, nor does it record the famous ‘centum/satem’ words for 100. In practice, many cognates stray in and out of given meanings over time, with the result that IE-CoR does in fact include c. 1600 cognate sets that go back to Proto-Indo-European, and have since spread across our 170 reference meanings: see https://iecor.clld.org/cognatesets?sSearch_3=Proto-Indo-European. For a full clarification of the nature and priorities of IE-CoR with respect to other types of language resources on lexemes and cognates, see [^8^SM3.5.2].

While there is extensive methodological debate on the pros and cons of different data types for researching language histories, qualitative linguistics has generally strongly favoured change in sound (phonology) and word structure (morphology)^3^. Computational phylogenetic analyses, however, have generally preferred cognacy data. Particularly for the task of estimating the time-depth of language families, Bayesian phylo-chronology has grounds for seeing cognacy data as by far the most viable and extensive data-type^15^. To this end, IE-CoR aims to provide a reference dataset for patterns of cognacy in 170 reference meanings, as the form of input data most appropriate and most valuable for state-of-the-art phylogenetic analysis software.

There are significant methodological challenges in coding language data as numerical or binary values. Most forms of language data are non-discrete, not measurable instrumentally, and meaningful only in the context of a wider system. This goes in particular for word meanings, and so forms a central challenge for a dataset like IE-CoR. Comparison is complex also because languages differ greatly in how they organise the relationships between the sound signal, word forms, and meanings. To overcome these difficulties, we implemented strict and comprehensive protocols to ensure data accuracy, consistency and comparability. These include in particular protocols to avoid inconsistency in the number of lexemes included per language, a known problem with previous datasets and which has led to known artifacts in past phylogenetic analyses^2^. Our protocols on all levels are set out in detail in the corresponding subsections under ‘Methods’ below.

### Background: cognate datasets

IE-CoR emerges from a long history of previous work across several fields within linguistics. Work to draw up cognate datasets, specifically, was begun above all to provide data for an earlier (and now largely abandoned) methodology of *lexicostatistics*, from the 1950s onwards^16,17^. Over the decades, much research went into methodology for compiling these datasets, by several independent research groups, e.g.^18–24^. IE-CoR draws in particular on^9,25,26^, and in some specific aspects on^20^.

That said, IE-CoR was developed independently of previous cognate datasets for Indo-European^19,24^, with data newly collected and coded, for a newly optimised set of reference meanings, and a new set of 160 Indo-European languages, many more than in previous datasets (see the comparison in Table 1 in^2^).

In methodology, too, IE-CoR also explicitly departs from existing approaches^19–21^ in multiple essential respects — indeed it needed to, in order to overcome various ongoing issues with past datasets. Firstly, IE-CoR does not adopt an existing ‘off-the-peg’ **set of reference meanings**, but optimises its own new set. That is, although sourced from within a combination of several widely used existing sets^16,17,20,27^, this was honed down by excluding many meanings that do not meet a set of optimisation criteria applied by IE-CoR (as detailed below under ‘Methods: Meaning sample’). For many of its reference meanings, IE-CoR builds on **meaning definitions** in previous proposals, but also adds its own different, more extensive and more targeted specifications. Protocols for both **lexeme and cognate determination** are likewise new, different, more extensive and more explicit [^8^SM3.5-3.6]. New protocols are necessary, and duly introduced, to resolve issues that led to dataset inconsistency^2^, in particular tolerance of multiple near-‘synonym’ lexemes^18,19^. In part, our approach takes further previous suggestions in this direction^20,21^, but in other ways marks a new departure from them too (as detailed below under ‘Methods: Lexeme determination’).

Another key issue raised in the extensive literature on cognate datasets is how to handle and encode loanwords. From a phylogenetic perspective, loanwords represent horizontal transfers, which if undetected or inappropriately coded can act as a confound to recovering phylogeny. IE-CoR introduces a new data structure for encoding loanwords and distinguishing between different types, another methodological innovation required to more accurately represent both horizontal and then renewed vertical transmission in the histories of loanwords. Again, although much previous methodological research (e.g.^18,19,22^) has engaged with this issue, past datasets have nonetheless handled loanwords essentially by just flagging that status, but within the same dataset structure as for cognates. Phylogenetic analyses have thus only been able to treat loanwords as singletons^5^, as singletons or as true cognates^6^, or as “lacunae”, i.e. missing data^7^. Given that all these approaches have weaknesses^2,28^, IE-CoR handles loanwords in a new way, now by a dedicated new relational structure between data tables in the dataset. This is articulated around the new concept of a loan *event*, to capture the resumption of vertical inheritance after such an event, and also establishes a necessary distinction between two different types of loan event (see below under ‘Methods: Loanwords’).

In sum, although naturally it builds on much past work on cognate datasets, IE-CoR is essentially new and of (much) greater scale in all main aspects of its **coverage** (languages, reference meanings), **methodology** (meaning definitions, coding protocols, structures for handling horizontal transmission) and **data** collection and coding (team members, and their lexeme and cognate determinations).

### Background: research in cognacy, etymology and lexical semantics

Compiling cognate datasets relies in turn on a wider research background in the subfields of linguistics that underlie this task in the first place. Two subfields are especially directly involved. To determine cognacy, the field of **etymology** (e.g.^29–31^) traces the historical origins of words through changes in sound (phonology), word structure (morphology), and meaning. To determine which lexemes most appropriately correspond to each other in meaning, from one language to the next, relies on research in **lexical semantics** (e.g.^32–35^). The latter field has developed the analytical approaches that are essential to navigate the semantic indeterminacy in how meanings overlap and shade into each other within and between words and languages.

We set out in more detail below, under ‘Methods: Cognate determination’ and ‘Methods: Meaning sample’ respectively, how IE-CoR builds on theoretical research in these highly complex fields. Here we briefly clarify more generally how IE-CoR stands within this background research context in linguistics, particularly given the practical, ‘big data’ imperatives in providing input to Bayesian phylogenetics, which needs very large-scale datasets from which to estimate all parameters most effectively.

IE-CoR cannot itself aspire to be a highly detailed work of **etymology**, when that task is often so highly complex, even for a single word. Many lexemes are made up of more than one meaning-bearing component (a ‘morpheme’, e.g. the three in the word *un*·*help*·*ful*), and each one, or each particular combination of them, can have its own history. To ensure that collating a large-scale dataset was practicable with the resources available, cognacy analysis was prioritised. In IE-CoR the etymology of each of its 25781 lexemes is in practice taken only as far as one basic comparative datum per lexeme: the cognacy state of only the primary root morpheme in that lexeme. To do this means first of all identifying which morpheme within a lexeme is its root; or, if there is more than one root, which of them can be considered primary, and on what grounds. This entails following certain analytical principles long established in linguistics. IE-CoR protocols take the root morpheme as the one that most directly bears the semantic content most specific to the IE-CoR target meaning; see below under ‘Methods: Cognate determination’. This limitation to encoding the cognate state of only the primary root morpheme (per language per meaning) means that IE-CoR does not aspire to a “perfect etymology”^31^ of all lexemes, and does not attempt to encode the cognacy or histories of any additional morphemes (e.g. affixes) other than the primary root morpheme. This focus helps to attain the essential dataset objective: *consistency* in encoding the specific data point in which all languages are compared in IE-CoR, i.e. the primary cognacy state in each meaning in each language.

IE-CoR likewise rests on background linguistic research in **lexical semantics**, particularly on the challenge of semantic indeterminacy. As an example, Swadesh^16^ listed one of his reference meanings as ‘hit’, but that English word is indeterminate between senses of either striking a physical blow or reaching a target (i.e. the opposite of *miss*). The dataset in^19^ entered two different corresponding German lexemes, *schlagen* and *treffen* respectively. Indeed it treated them as if ‘synonyms’, even though in German they are not, and simply reflect the indeterminacy of the English word. Other datasets drop this meaning entirely^17,20^, while IE-CoR’s solution is to pin down more narrowly the target sense for lexeme determination, as set out in the full IE-CoR definition at https://iecor.clld.org/parameters/hit. (This explicitly limits the IE-CoR reference meaning to the sense of striking, ensuring that lexeme determination in German can unambiguously exclude *treffen* and confirm *schlagen* as the primary lexeme.)

Yet as with etymology, IE-CoR’s priority is to create a big data resource, not to be a work of detailed theoretical analysis in lexical semantics. Indeed, while the wider background context in linguistics recognises all too well the known scope for complexity in principle in both etymology and lexical semantics, the discipline nonetheless continues to view the task of drawing up cross-linguistic cognacy datasets as essentially viable in practice. Leading science journals regularly publish language family phylogenies based on new cognate datasets (e.g. on the Sino-Tibetan language family^36–38^). For Indo-European, which is comparatively well researched and documented, the task is equally viable. The complexities of lexical semantics across languages are well known to our linguist coding team, informed by the insights from decades of research in this field, not least the formal and quantitative perspectives now available from major datasets on related issues, such as colexification (e.g.^39,40^). By design, therefore, the IE-CoR project workflow involved a major, iterative process to minimise the obstacles from semantic indeterminacy, by adopting methodological solutions on multiple levels, as set out below under ‘Methods: Meaning sample’. In practice, those solutions did make it feasible to determine primary lexemes and cognate states across different languages to a high level of consensus in the vast majority of cases, sufficient to be viable for quantitative and Bayesian phylogenetic purposes^2^.

## Methods

### Overview of methodology

The IE-CoR dataset documents, for each of the 160 Indo-European languages it covers, which lexeme in that language corresponds to each of our 170 comparison meanings. For each meaning IE-CoR also groups together, into cognate sets, any of these lexemes in different languages that descend from the same shared ancestral wordform, e.g. English *foot*, German *Fuß* and French *pied*, which all descend from the Proto-Indo-European root *ped- (see the Definitions box).

Drawing up the IE-CoR dataset thus involved several different steps. First, we identified the languages to include in the dataset (Language sample). Second, we carefully delimited the reference meanings for which we would collect lexemes (Meaning sample). Third, we identified the primary lexeme in each language corresponding to each meaning (Lexeme determination). Fourth, we annotated the historical relationships between the lexemes in the dataset (Cognate determination). Each of these steps entails quite distinct methodological considerations which are discussed in separate subsections below. However, we first provide a general overview of how we designed and populated the dataset.

For IE-CoR, we aimed for a **language sample** that is extensive and well-balanced across both space and time for all 13 major documented clades of Indo-European, and their main subclades. The project coordinators worked with specialists in individual clades to identify candidate languages, then approached experts in these languages, inviting them to collaborate in developing the dataset. As a result, language coverage in IE-CoR is much wider than in previous datasets (see the comparison in Table 1 in^2^) and developing the dataset was a genuine collaborative effort, with input from 89 experts across all clades of Indo-European.

Drawing up a **meaning sample** presents considerable difficulties. For a dataset of lexical cognacy, such as IE-CoR, the frame of reference is ultimately semantic, in that lexemes in different languages are identified with each other because they share certain semantic content. Methodological difficulties arise because meanings are not fixed, either within any one language or across different languages. Meanings are ultimately indeterminate^41^ and languages differ in how their lexical (and grammatical) forms express semantic content and map across semantic space with respect to each other. These difficulties can be mitigated, however, and much of the IE-CoR workflow was structured to this end. In 2016 and 2017, we invited experts from across Indo-European (and other language families) to workshops in Jena, Germany, specifically to establish consensus across the team on the set of IE-CoR protocols to be followed in data collection. Involving language specialists in this design phase, in particular in establishing the reference set and in drawing up meaning specifications, ensured that many risks of inconsistency between coders and other potential methodological obstacles, discussed further in the following subsections, were resolved in advance.

The first phase in populating the dataset was **lexeme determination**. For each of the 160 languages covered, one or more experts, from among the IE-CoR consortium of 89 language specialists, determined which of that language’s lexemes is the primary term for each of the 170 IE-CoR reference meanings, following rigorous protocols guiding data collection (see below under ‘Lexeme determination’), to identify consistently across languages which lexeme to take as ‘primary’.

The second phase in populating the dataset was **cognate determination**, i.e. aggregating into cognate sets those lexemes in different languages that descend from the same single wordform at an earlier, common ancestral stage. In the meaning foot, for example, French *pied*, German *Fuß* and English *foot* are all cognate, because all derive from *ped- (*pod-) in Proto-Indo-European (cognate set 225 in IE-CoR). In the meaning black, however, French *noir*, German *schwarz* and English *black* are not cognate, but descend from different sources (see cognate sets 858, 188, and 1343 respectively, in the meaning black in IE-CoR).

Cognate determination was performed by an expert team of historical linguists, making full reference to the extensive research literature on the historical and comparative grammar and etymology of the Indo-European languages. Strict protocols were followed to deal with potential obstacles to cognate determination that arise from various types of linguistic phenomena: compounding, suppletion, parallel derivation (a form of homoplasy), ideophony, and above all the horizontal transfer of loanwords, particularly frequent in language histories. (On IE-CoR structures and protocols for these issues, see below under ‘Cognate determination’.)

### Language sample

IE-CoR provides comprehensive sampling of historical varieties of Indo-European, and balanced coverage of all 13 major clades and their main subclades. Of the 160 IE-CoR languages, the 52 historical (i.e. not present-day) varieties are of particular value for exploring evolutionary processes within the Indo-European language family. This is because the time-frames during which these language varieties were spoken and recorded provide calibration ranges, from which rates of change can be more robustly estimated, as they vary widely by language, meaning and through time, notably by Bayesian phylogenetic inference^15^. In some cases, such as Tocharian A and B, the languages included in the IE-CoR dataset are the only well-attested taxa from a deeply divergent clade that is now long extinct. We aimed to achieve extensive coverage of the earliest well-attested languages within each main clade.

Historical language varieties present particular problems for data collection, however, as the surviving data sources are not always extensive enough to allow lexemes to be securely determined in all 170 IE-CoR reference meanings. In some cases, a given meaning may simply not appear in the surviving text corpus, so no lexeme for it is known. In other cases, attestations may not be extensive enough to decide reliably between potential alternative lexemes. IE-CoR policies for consistent data collection thus include additional protocols [8: SM3.5-3.6] for handling such difficulties specific to historical varieties. Attestation is uneven also for certain modern languages, although for different reasons (see below).

Once all branches and sub-branches of the family are represented, and sampled in as balanced a way as possible, adding further language varieties very close to others already in the dataset brings fast diminishing returns on the resources required to analyse and code them. They contribute almost no additional discriminatory phylogenetic signal (or data on rate and date estimations) between major branches in the family’s deep tree structure, the primary research objectives for a dataset such as IE-CoR. To avoid such oversampling, language varieties were not generally covered in IE-CoR if they could safely be predicted to be less than 4% different to any other language already in the dataset (i.e. a threshold of at least 7 out of our 170 meanings). Exceptions were permitted for some standard languages with major competing varieties, such as both Bokmål and Nynorsk standards of Norwegian (2 differences), or European vs. Brazilian Portuguese (6 differences).

A disproportionate focus on the literary languages of the global North is known to bias research results in cross-linguistic research^42^. Although some reliable reference materials are available for lesser-studied Indo-European languages, previous cognacy datasets have tended to undersample such languages^8^. IE-CoR takes steps to attenuate this bias, by extensive sampling of the Nuristani and Indic languages of the Hindu Kush, non-standard Iranic languages, and minority and regional languages in Europe.

### Meaning sample

The meanings in the IE-CoR reference set are a sample of the core lexicon of a language, e.g. the lower numerals, adjectives such as colour terms, nouns such as the names of parts of the body, and basic verbs such as drink and sleep^32^. A well known and prominent problem in lexical semantics is that of indeterminacy^41^ (see under Background above), so a primary concern for the IE-CoR project was to draw up a set of reference meanings that would nonetheless facilitate consistent and reliable data collection as far as possible. Indeed, the first phase in the IE-CoR project workflow was structured to this end.

The IE-CoR meaning set began out of a wider set of candidate meanings drawn from three existing sets: the Leipzig-Jakarta 100 set that emerged from the World Loanword Database^39^, and the original Swadesh-100 and Swadesh-200 sets^16,17^. These sets overlap considerably and together make for a combined candidate set of 235 meanings.

At the IE-CoR workshops in 2016 and 2017, each of the 235 candidate meanings was evaluated in detail by a consortium of experts on languages across the branches of Indo-European (and other families), against an agreed set of criteria drawn up to eliminate as far as possible any scope for inconsistency in data collection and coding. We set out these optimisation criteria in detail below, but to first summarise briefly, they favour meanings that are: as universal as possible (at least across Indo-European); amenable to the most consistent lexeme and cognate determination; and not overly susceptible to parallel derivation or horizontal transfer. This process narrowed down the 235 candidate meanings into the final 170 meanings of the IE-CoR reference set.

Optimisation criterion 1 is universality: IE-CoR avoids meanings that are geographically or culturally specific, or where there are particularities in the English term used as the meta-language for labelling reference meanings. Most IE-CoR meanings are expected to be universal to human cultures and contexts, e.g. water, moon or eye, and in many cases correspond to basic-level categories in prototype semantics^35^, e.g. bird and dog. A handful of IE-CoR meanings, such as red and snake, are effectively universal across Indo-European languages, even if not in all human languages. Only very occasionally was an IE-CoR meaning ultimately found to be inapplicable to a given language covered in the dataset. Kumzari, for example, spoken in the arid Arabian peninsula, has no straightforward lexeme for the meaning lake. One consequence of this universality criterion is that the IE-CoR meaning set consists mostly of relatively concrete nouns and verbs, more so even than its predecessor sets (e.g.^16,20,27^). Meanings that serve grammatical functions, such as IF or because, failed to meet an ancillary criterion of being universally expressed by content lexemes: many languages express such meanings through syntax or morphology rather than by a dedicated standalone lexeme such as English *if* or *because*. So while the candidate meaning sets from which IE-CoR optimised its own set had themselves aspired to (near-)universality, perspectives from the diverse set of IE-CoR languages and linguist authors nonetheless revealed problems with the (non-)universality of some candidate meanings, hence their exclusion.

Optimisation criterion 2 is amenability to consistent lexeme determination. Meanings should be maximally unambiguous, identifiable, amenable to an absolute definition applicable across all language taxa, and not tied to individual lexemes in English (as the meta-language used to label the meanings). The lower numbers, for example, do not permit gradience and are thus ideal in this regard, but with many other semantic fields, languages differ in how they divide them up. There are considerable differences in the scopes of the lexemes that languages use to describe colour or temperature, for example^33,34^. Many other languages do not have lexemes that overlap neatly with English *cold* and *hot*, for instance (whose usage is also a function of how English uses its own further terms *cool* and *warm*). Each IE-CoR meaning definition therefore provides extensive, detailed specification of the narrowed-down sense and context that is to be targeted in lexeme determination, to ensure that the primary lexemes identified in all languages correspond to each other as accurately as possible. Some meanings from the candidate set were excluded as being too closely defined by the English lexeme used as a label. stab, for example, rarely corresponds straightforwardly to a single lexeme in other languages. rope, meanwhile, fits into a segmentation of semantic space that is highly specific to English, where the lexeme *rope* contrasts — in particular and somewhat idiosyncratic characteristics of thickness and function — with *string*, *cord*, *line*, *thread*, etc. Both stab and rope are therefore among the meanings excluded from the IE-CoR 170 set on our optimisation criteria.

As well as being amenable to consistent *lexeme* determination (criterion 2), the IE-CoR meaning set was optimised to be amenable also to maximally consistent *cognate* determination, our optimisation criterion 3. Cognacy cannot be considered a property of full words, but of morphemes (i.e. minimal, discrete meaning-bearing units, see the Definitions box). So in lexemes made up of multiple morphemes, each morpheme has its own cognacy state. This introduces potential for uncertainty and inconsistency in determining cognacy in multi-morpheme lexemes. Recent work has tried to address this problem^43–45^, but in IE-CoR, cognacy in such cases is by default determined for whichever of the component morphemes is the root morpheme of the whole lexeme (see below under ‘Cognate determination’). It is usually fairly trivial to identify which morpheme is the root morpheme, at least for meanings that have relatively concrete referents. In meanings that serve grammatical functions, however, lexemes are often composed of multiple morphemes without a single clear root. In the candidate meaning what?, French *qu’est-ce que …?* consists of no less than four morphemes (literally ‘what is this that …?’). Similar complexities arise in many languages’ equivalents for because (the English lexeme *because* is itself formed historically of two morphemes, literally *by* and *cause*). The (grammatical) nature of both of these candidate meanings frustrates consistent cognate determination in many languages, and both were therefore excluded from the IE-CoR 170 set.

Optimisation criterion 4 excluded meanings highly susceptible to parallel derivation. Again, meanings that serve grammatical functions typically pose problems on this level too. Meanings such as because and what?, as well as deictics — such as this, that, here, there, and personal pronouns — are particularly liable to being repeatedly restructured out of the same inherited morphemes. In French, for example, *par∙ce que* (because) and *qu’est-ce que?* (what?) roughly equate to ‘for this that’ and ‘what is this that?’, creating complex and only partial overlaps in cognacy with new formations in other Romance languages, even when they retain (some of) the same Latin source forms. That is, the individual component morphemes go back to the proto-language, but the particular restructurings and combinations of them do not. These complexities are amplified when assessing deeper Indo-European cognacy with other branches. Lexemes for because show similar complexities across Slavic languages, for instance. Such cases represent complex combinations of partial cognacy states with indeterminacy across multiple possible analyses of cognacy status, making it difficult or impossible to establish definitive, consensus codings.

Optimisation criterion 5 excluded those candidate meanings most susceptible to horizontal transfer, i.e. meanings in which inherited lexemes are often replaced by others borrowed from other languages. Specifically, IE-CoR is a dataset of cognacy, as a record of preserved phylogenetic signal. Loanwords, however, are cases where that is replaced by horizontal signal instead, and are thus confounds to phylogenetic signal. Loanwords also knock out vertical phylogenetic signal in biased ways, structured by socio-cultural and geographical factors independent of phylogeny. Established reference meaning sets for cognate data generally aspired to avoid meanings assumed (qualitatively) to be highly borrowable, but published statistics are now available. The large World Loanword Database (WOLD)^39^ provides empirical cross-linguistic data on the relative “borrowability” (or more strictly, susceptibility to borrowing) of candidate meanings^27^. On that quantitative basis, IE-CoR excluded meanings found to contain the most horizontal, and thus the least vertical, phylogenetic signal. The empirical WOLD statistics identify the candidate meaning person, for example, as relatively frequently borrowed. An initial test survey confirmed this in practice also for our Indo-European language set. Of the 165 lexemes for person across all IE-CoR languages, 29 arose out of 10 independent loan events, and a further 34 out of independent, parallel loans of the same source lexemes. These horizontal transfers affected 6 out of the 13 main Indo-European clades, confirming the poor retention of vertical signal in the meaning person, and the limited utility of retaining it in our optimised meaning set. person was indeed among the candidate meanings excluded from IE-CoR on these grounds.

Finally, optimisation criterion 6 was applied to the set of reference meanings as a whole, to strike a balance between meanings for which lexemes across the IE-CoR language sample fall into a smaller or a larger number of cognate sets. In some meanings, such as the lower numbers, inherited lexemes tend to be very highly stable (at least in the IE-CoR language taxa), so cognate status varies very little in IE-CoR. In the meaning five, for example, all lexemes in the 160 languages covered fall into the same single cognate set, defined by their shared origin in Proto-Indo European *pénk^w^e (see five in IE-CoR). Other meanings show much more variability: in the meaning dirty, for example, the 148 lexemes attested for the 160 IE-CoR languages fall into as many as 81 cognate sets. Meanings in which cognate status is less variable are essential to preserve signals on deep relationships, while meanings in which cognate status is more variable provide higher resolution on shallow relationships and faster change over time. Many previous datasets [^7,20,46^see 2] have lost this finer discriminatory signal by reducing the reference set to 100 meanings or fewer, targeting only those with the least variability. IE-CoR does not lose this resolution, and retains a balance between meanings with lesser and greater variability in cognacy.

To label each reference meaning, datasets of lexical cognacy have from the outset relied on lexemes in English, a few of them provided with “parenthetic additions” to narrow down the intended sense at least to some degree, e.g. “bark (of tree)” or “earth (soil)”, or in some cases actually to extend it, e.g. “berry (or fruit)”^16^. More recent research has extended the “attempt at semantic specification”^20^, although for the reasons set out below under “Meaning definitions”, IE-CoR had to take this much further still. Each of the 170 reference meanings in the IE-CoR set has been given a new, extensive definition and narrow specification of the target sense intended in IE-CoR. See also below for a sample IE-CoR meaning specification for the meaning fire.

### Lexeme determination: Overview

The first main stage in data collection, performed by a consortium of 89 language experts from 2016 onwards, was lexeme determination. This involves identifying which lexeme in each language is its primary term for each IE-CoR reference meaning, i.e. which most accurately meets the targeted IE-CoR meaning specification. To best assess this, IE-CoR engaged a linguist with a specialisation in that language: for modern languages, usually either also a native speaker, or working closely with a knowledgeable native speaker; for historical languages, an expert in the historical linguistics of that language. In some cases, the same linguist collected data for multiple, usually closely related languages, and for most languages more than one expert collaborated on data collection and revision.

To guide and ensure the most consistent possible lexeme determination across different languages and linguist coders, IE-CoR introduced explicit protocols for lexeme determination. (These protocols are also integrated case by case into the extensive new definition of each reference meaning; see below under ‘Meaning definitions’.) The core concept is that of the single, **primary** lexeme in a given language, and the IE-CoR protocols set out explicit criteria to define and evaluate ‘primary’ on multiple linguistic levels, particularly frequency, register, morphosyntactic variation and paradigms. These protocols specify, for instance, that for a lexeme to be considered primary in a given meaning, it should be usable in a broad range of contexts, belong to the default register of the language, be literal rather than figurative, and be learnt earlier rather than later during native language acquisition. The full set of protocols for lexeme determination is set out extensively in [^8^: SM3.5]. Again, it is linguists and native speakers who are best placed to assess which lexeme these protocols single out in any one language.

Since these protocols, criteria and detailed definitions of reference meanings are specific to IE-CoR, the *source* of our lexeme data is thus the IE-CoR author(s) for each language, as author(s) of the lexeme determination decision. No published generic dictionary resources are specific to the IE-CoR criteria. Rather, to provide data to clarify and support a particular lexeme determination, where appropriate, the IE-CoR data record for a lexeme also includes an additional Notes field. This can be used in particular to mention other near-synonym lexemes and the criteria followed in evaluating them, as for instance in the Note on lexeme iecor.clld.org/values/73-35-1. The Note on 177-33-1, meanwhile, lists the individual attestations in the corpus of New Testament Greek that support that IE-CoR lexeme determination. Another field can cross-reference and link to authoritative online resources, where available, on any individual IE-CoR lexeme, e.g. the English lexeme *fire* at 22-50-1 links to the Oxford English Dictionary entry www.oed.com/dictionary/fire_n, while French *feu* at 25-50-1 links to www.cnrtl.fr/etymologie/feu.

### Lexeme determination: Synonymy

One particular and well known issue with cognate datasets is synonymy. This arises when, for a single meaning in a given language, one might enter not just the usual single lexeme, but more than one lexeme with (very) similar or overlapping meanings, i.e. (near) synonyms. For the meaning small, for example, for English one might enter not just the lexeme *small* but also *little*.

In lexicostatistics, various approaches allowed and even encouraged entering near synonyms (see^18,19^), and explicitly set out how to handle them in calculating lexicostatistical scores of cognate overlap between any pair of languages, collected into a triangular distance matrix. Synonyms are in practice handled very differently, however, when cognate data are fed instead into (character-based, not distance-based) phylogenetic models of cognate evolution over time^2,28^, especially those that require input data in binary format. This calls for a new approach to synonymy when devising datasets now for this very different application.

Synonymy usually refers to two or more *lexemes* in a *single* language, in a given meaning (e.g. *small* and *little* in English). One past approach has used ‘synonymy’ in a different sense, however: to refer to cases where two or more *cognate sets* are found across a *set* of related languages. Attempting lexeme determination for an unattested proto-language^21^ makes it necessary to try to identify, among any such competing cognate sets across its daughter languages, which was the original primary lexeme at the proto-language stage. IE-CoR deliberately avoids any such attempts to code hypothetical proto-languages as actual data languages in its dataset, however, a task widely seen as unreliable and potentially circular^2,3^, so this specific interpretation of ‘synonymy’ in^21^ is not relevant to IE-CoR’s task.

Closer to the IE-CoR approach to reducing synonyms is the “attempt at semantic specification” of the Swadesh 100 meaning reference list in^20^, although that methodology was still intended primarily for applications in lexicostatistics. With the switch to phylogenetics, however, came an unsuspected consequence of the synonyms entered in cognate datasets drawn up originally for lexicostatistics, not realised^2^ until several years after major phylogenetic analyses of Indo-European^5,6^. In the “IELex” dataset on which both were based^24^, although most data points had just a single lexeme entered per meaning, in a significant proportion of the dataset *more* than one near-synonym lexeme had been entered for the same meaning in a single language. Lexicostatistics generally counted a ‘match’ per language pair if any such ‘synonyms’ were cognate, without any significant further consequences. Most Bayesian phylogenetic models, however, take input data expressed as *binary* characters. Each cognate set is thus taken as a character with states of either present (1) or absent (0). In these terms, for a given reference meaning, generally only a single lexeme (and thus cognate set) is ‘present’ (state 1) — but wherever a near-synonym was also entered, more than one cognate set is thus also ‘present’ (one for each near-synonym lexeme). The phylogenetic models do not register any linguistic concept of synonymy, however. Rather, the cognate sets of the two (near synonym) lexemes constitute two entirely different data characters, both ‘present’, and the models work in terms of *changes* over time from a state of 0 to 1 (or *vice versa*). Any differences in how many cognate sets are present (i.e. any near-synonyms) can thus be represented only in terms of additional *changes*. So wherever a Language A is coded with only a primary lexeme, but Language B with any near-synonyms, then the model will infer *more changes* in Language B than in A — even when that is not the nature of the linguistic difference, but just an ‘excess’ from inconsistently coding more cognate sets in one language than in another. In Bayesian phylogenetic outputs based on such data^5,6^, these inconsistent excess data characters led directly to conspicuous artefacts. Excess changes and thus branch lengths, and the resulting inconsistency for different languages and in different parts of the trees, undermined the estimated chronologies, as identified only recently in^2^.

In short, dataset inconsistency in synonyms, while relatively innocuous for lexicostatistical counts, becomes critical when data are binarised as input to phylogenetic analysis models. To that end, a new input dataset was needed, drawn up to new and more stringent protocols specifically to limit synonymous entries, as applied in IE-CoR, and going beyond past methodological proposals for datasets for lexicostatistics (including^20^). IE-CoR’s set of protocols for lexeme determination, discussed above, were targeted not least towards this essential task of eliminating any scope for ambiguity between near synonym lexemes, so that in all languages coders could unequivocally identify the single primary lexeme in as many as possible of the IE-CoR reference meanings. The same protocols were also essential in guiding how IE-CoR devised its narrower, targeted meaning definitions (see next section) to the same essential aim of maximum consistency in lexeme determination — again, not least by eliminating scope for synonymy. Together, these new IE-CoR approaches made it viable in practice to apply a strict limit on tolerance for multiple (‘synonym’) lexeme entries per meaning to under 4% of meanings for any one language. IE-CoR thus achieved very high dataset consistency in this respect, with an average synonymy of just 1.21%), as set out in detail in [^8^: SM1.4] and illustrated in [^8^: Fig. S1.4], contrasting with the versions of the IELex datasets used in^5^ (average 13.87%) and^6^ (average 15.32%).

### Lexeme determination: Meaning definitions

Every IE-CoR reference meaning was given a new and dedicated definition, to specify the particular sense targeted by IE-CoR in lexeme determination, devised especially to help eliminate scope for synonymy. In fact, many instances considered synonymy in past research are not actually cases of synonymy at all, but of failing to specify sufficiently — among the various senses of a polysemous English word used to label a reference meaning (such as hit) — which of those senses was to be targeted in lexeme determination. As explained in “Background” above, Swadesh’s meaning hit in^16^ was not given any “parenthetic addition” to narrow it down, and the dataset^19^ duly entered two lexemes in German, *schlagen* and *treffen*. Focusing the reference meaning only on the sense of striking a physical blow, however, and not on that of reaching a target (a secondary sense of English *hit*), resolves this ‘synonymy’ and leaves the primary lexeme unambiguously determined in German as *schlagen,* not *treffen*.

Devising the new IE-CoR meaning specifications and explanatory contexts for each of the 170 reference meanings was an integral part of the project workflow. The IE-CoR workshops in Jena in 2016 and 2017 served to explore and reach consensus across consortium members on which new specifications ensured the most unambiguous and consistent lexeme determination across all languages. The IE-CoR team went through all reference meanings one by one, comparing their complementary perspectives from all branches, to identify and resolve potential difficulties in semantic specifications, to circumscribe more narrowly the sub-sense to target in each case, and to devise a suitable, viable formulation and example context for each. In this, IE-CoR could build on parts of some previous definitions in^20^, also continued into IELex^24^, for about half of the IE-CoR reference meanings (although not for those explicitly excluded from IE-CoR by its optimisation criteria). Nonetheless, IE-CoR’s aim to provide an input now for Bayesian phylogenetics brings a heightened imperative of limiting near-synonymy, to avoid the artefacts that it causes (see under ‘Lexeme determination’ above). With respect to published cognate datasets devised with methodologies to serve lexicostatistics, then (including^20^), all IE-CoR meaning definition texts are new: different, more explicit, more targeted and thus far more extensive, as can be seen from the long example definition of the IE-CoR reference meaning FIRE below.

In particular, these new definitions are devised in line with, and cite, the new IE-CoR protocols for lexeme determination, set out in full detail in [^8^: SM3.5]. IE-CoR definitions are framed to pinpoint a specific target sense in every case, as the particular data point in which all languages in the dataset are compared. In many cases, the English lexeme used as the label for a meaning shows polysemy, as with hit discussed above. Each definition therefore invokes specific IE-CoR protocols to narrow down to a more specific sense and usage context, and to explicitly exclude other senses within any significant polysemy in the English label lexeme. Furthermore, while^20^ provided two or usually three different example context sentences per meaning, the IE-CoR protocol is the opposite: to focus on just a single sense and illustrative context sentence, to avoid opening scope for near-synonymy.

All IE-CoR meaning specifications can be consulted on the web application, e.g. at https://iecor.clld.org/parameters/fire for fire, also reproduced below.

### Cognate determination

The second main stage in data collection and analysis was cognate determination. Lexemes in different languages that descend from the same ancestral word form in a common ancestor language are termed **cognates** (see Definitions box). For each meaning, cognate lexemes were grouped into cognate sets on the basis of their shared ancestry. It is not always straightforward to categorise lexemes into mutually exclusive cognate sets, as there are different forms or levels of cognacy, including partial cognacy, i.e. between some but not all morphemes in a word, as we shall see below. Also, different specialists can hold conflicting hypotheses on the origins and cognacy of individual lexemes. IE-CoR follows the main established reference works^47,48^ wherever possible and aims for a consensus view, where a majority of authoritative reference works agree on (root morpheme) cognacy between lexemes in different languages.

For example, in the meaning fire, all lexemes in the set IE-CoR 219 are unanimously agreed to continue the Proto-Indo-European heteroclitic noun *péh_2_-u̯r̥, *ph_2_-u̯én- ‘fire’. In some cases, authoritative handbooks agree that the lexemes included in an IE-CoR set are indeed cognate with each other, even though there may remain some disagreement on the precise details of the reconstruction. In the meaning name, for example, IE-CoR cognate set 82 (with IE-CoR reference form *Hneh_3_-mn̥) there is consensus that all the lexemes are cognate, but the authoritative reference works disagree over the specifics of the reconstruction of the laryngeal at the start of this proto-form (symbolically transcribed H) and alternations in root vocalism. (It is inherent in the form of the derived lexemes as they are first attested that they are open to opposing interpretations of their earlier sound-change histories).

Generally, IE-CoR has followed the practice of taking an etymology as far back as consensus view agrees, across the established reference handbooks. Deeper etymologies are not always agreed upon in the literature, and in these cases cognacy judgements are normally taken only as far back as that common point of agreement. In the meaning bad, for example, on one view Proto-Albanian *kakii̯ā̆- (IE-CoR 6597) and Proto-Greek *kakó- (IE-CoR 7332) may go back to a common inherited root *kak-^49^. Nonetheless, various difficulties and uncertainties attend that hypothesis, and other specialists are reluctant to accept this etymology^50–52^, so the broader consensus view followed in IE-CoR is to keep these cognate sets separate, but to register in the meta-data for those cognate sets a cross-indexing to each other, as a non-consensus cognacy judgment *proposed* by some analysts. These meta-data ensure that the IE-CoR dataset can also be used to run re-coded analyses to test the phylogenetic implications of these competing proposals on cognacy.

For a detailed description of the workflow for determining consensus views in IE-CoR cognacy judgements, see^53^. For individual languages, cognacy determination relied also on the input of specialists in the historical linguistics of each language. Furthermore, we implemented dedicated protocols for cases where cognate determination is not straightforward: with lexemes formed of more than one meaning-bearing unit (‘morpheme’); in instances of suppletion; and in rarer cases of ideophony and of parallel derivation (one form of homoplasy in cognate datasets). We also implemented a series of novel protocols to handle cases of horizontal transfer. We now look at each of these types of case in more detail.

Many lexemes are composed of more than one morpheme: one root morpheme plus one or more affixes, or indeed two root morphemes (e.g. compound nouns and light verbs), for instance. (As standard reference works on the linguistic analyses of such aspects of word structure, see for example^54,55^.) For such morphologically complex lexemes, cognate determination is based on the morpheme whose meaning is more specific to the target meaning.

In lexemes with *only one root* morpheme, it is invariably that root morpheme, not any affixes, whose meaning is more specific to the target, so cognacy is determined on the basis of that single root. For the meaning breathe, for example, with Modern Greek *anapnéō* ‘breathe’, determination of cognacy is based not on the prefix *ana-* ‘re-, up’, but rather on the verb root *pné*- ‘blow, breathe’.

When a lexeme is made up of *more than one root* morpheme, cognate determination is based on whichever of those roots bears the most specific semantic content. The English word *yesterday*, for example, is composed of two roots: *yester* and *day*. Of these, it is *yester*, not *day*, that is more specific to the target meaning, so in IE-CoR the primary cognacy state in this meaning in English is that of the morpheme *yester*, which indeed is cognate with the root morpheme in German *gestern* in the same meaning.

Similarly, in the meaning fear (as a verb), German *Angst haben* has two roots, literally ‘fear-have’, but the verb root *haben* is semantically weak here, and the meaning specific to fear is borne by the noun root *Angst*. The primary cognacy state in German for this meaning is thus determined for the root morpheme *Angst*, not *haben*. (Note that although Swadesh chose fear as the label for his reference meaning, English no longer uses that simplex verb as its primary lexeme, but multi-root lexemes like *be scared*, *be afraid* or *be frightened*, with differences between them in register and regional usage).

Some languages make particularly frequent use of constructions like German *Angst haben*, i.e. many of their lexemes for verb meanings consist of a combination of a noun (or adjective) plus a common verb, such as Modern Persian *yax bastan* ‘freeze’, literally ‘ice close’. Again, in IE-CoR cognacy is determined on the basis of the component semantically more specific to the meaning freeze, i.e. *ice*, rather than the semantically more general *close*. Or to return to the meaning breathe illustrated above, while Modern Greek has just one root plus affixes, examples with two root morphemes are Persian *nafas kešidan* and Scottish Gaelic *tarraing anail*, both literally meaning ‘pull breath’. Here cognacy is determined in IE-CoR on the word for ‘breath’, i.e. *nafas* and *anail* respectively, much more specific to the target meaning breathe, not on the semantically more general words for ‘pull’.

Multi-morpheme words are a much discussed issue in etymology (see under ‘Background & Summary’ above). In IE-CoR, the etymology of a lexeme is in effect taken only as far as identifying the root morpheme that carries most directly the semantic content most specific to the IE-CoR target meaning, and assigning that root morpheme to a cognate set (or in the case of a loanword, by IE-CoR’s equivalent structures for handling horizontal transmission — see below). Strictly, then, IE-CoR only aspires to encode and compare languages for the cognate status of the *root* morpheme of the primary lexeme in each language. The full etymology of an individual lexeme, including other component morphemes, can generally be consulted through the extensive referencing that accompanies a cognate set in IE-CoR, but lies beyond the level of cognacy encoding aspired to (and feasible, for that matter) in IE-CoR, for reasons of practicality and indeed cross-linguistic consistency. In many cases, multiple languages share the same root morpheme in a given meaning, but vary greatly in combining it with one or more other non-root morphemes, particularly suffixes in Indo-European languages. IE-CoR did not aspire to code the mass of far more complex cognacy relationships between such additional suffixes in its 25781 lexemes. This focus was essential to ensure that the dataset was a feasible objective in practice, within the resources and time available to the project. Indeed, limiting cognacy coding to this level means that it could continue to be viable even for words for which a “perfect etymology”^31^ may not be possible at all. So in cases of “partial cognacy”, i.e. where lexemes from different languages are cognate in one of their morphemes but not in all, the IE-CoR approach is to code lexemes as cognate only if the primary root morpheme is cognate, not when there is cognacy only in a non-root affix. See for example the discussion in [^8^: SM7.6.2.1] of the all meaning in^3^, or one approach to ranking partial cognacy in^25^.

Also for practical purposes of ease of identification, each cognate set is labelled with an “IE-CoR reference form”. This is usually the oldest traceable form of the root morpheme, whether in Proto-Indo-European itself, or as reconstructed just to one of the later, intermediate proto-language nodes of the family’s branches. In other cases, the reference form is limited to the single language that a root is found in (e.g. English *kill*), or with loanwords, to the form of that word as it entered a language, through a loan event.

A single lexeme stem, such as the English verb stem *eat*, is often the basis of a paradigm, or set of variant forms: e.g. *eat*, *eats*, *eaten*, *eating*. ‘**Suppletion**’ refers to cases where one of these variant word forms in a given verb or noun paradigm is not recognisably related to the others. In English, for example, *going* and *gone* are recognisably related to the stem *go*, but *went* is not, i.e. it is suppletive. (Cognacy is in this sense ‘partial’ across the paradigm as a whole.) To ensure that a dataset compares like with like between languages, the word forms compared must correspond not just to the same meaning, but also to the same position in the paradigm. In IE-CoR, to ensure consistency between all languages, the positions considered in cognate determination are strictly specified: for verbs, the present indicative third person singular form; and for nouns and adjectives, the nominative singular form (if morphological cases exist in the language at all). For the verb meaning GO, for instance, IE-CoR maps the French present third person singular form *va* (‘goes’) into the same cognate set as the equivalent Spanish lexeme *va*. That is, cognacy is not determined here on the basis of usual dictionary citation forms, i.e. the corresponding infinitives *aller* and *ir*, which are not cognate. (In principle another position in the paradigm could have been selected instead, so long as consistency was maintained. The IE-CoR team settled on its consensus conventions after discussion of various linguistic considerations: e.g. frequency, and the decline of the infinitive in the Celtic branch and in the languages of the Balkan *Sprachbund*.)

The lexemes in a cognate set descend with modification from a common ancestral form. However, lexemes may also be ideophonic, i.e. they may originate instead in ‘recoinings’ or alterations of particular sounds taken to be (auditorily) symbolic of that particular meaning. Alternatively, they may derive from the same inherited ancestral elements, but independently and in parallel in different clades after they had already split from each other. (In phylogenetic terms, such parallel derivations are instances of one form of homoplasy^2,56^.) The IE-CoR dataset includes boolean fields to identify cases of ideophony and clear parallel derivation — although used only where strict criteria were met, to exclude alternative possible origin histories. In the IE-CoR 1.2 dataset, only 5 clear instances of ideophony and 1 of parallel derivation met these criteria. The number of cases of parallel derivation is so low also because the IE-CoR meaning set optimisation policy, as set out under ‘Methods: Meaning sample’ above, excluded meanings that exhibit various well known cases of parallel derivation in Indo-European, e.g. with deictics.

In IE-CoR, each cognate set is, for practical purposes, limited to lexemes that correspond to the same IE-CoR reference meaning (this is inherent in defining the specific point of comparison between all languages in terms of that reference meaning). Given semantic shift, reflexes of the same underlying cognate set, i.e. lexemes that go back to the same ancestral form, may and indeed often do end up being used in different meanings in different languages. The Italian verb *vedere* and German *wissen*, for instance, both go back to the same Proto-Indo-European form *u̯ei̯d-, so strictly they are cognate. They have come to have different meanings, however: *vedere* means see, but *wissen* means know. They thus correspond to different IE-CoR meanings, and for more straightforward data handling, IE-CoR keeps cognate set records separate for each meaning. So *u̯ei̯d- for the meaning see is IE-CoR cognate set 929, whereas *u̯ei̯d- for know is 2307. Each meaning can thus be taken as a single multi-state variable, in which each state (i.e. each cognate set used in that meaning) is unique to that meaning. Nonetheless, to be useful as a data source on cognacy also *across* such semantic differences, IE-CoR also implements a concept of a cognate ‘superset’. IE-CoR cognate sets 929 and 2307, for example, are thus cross-referenced to each other within cognate superset 304 *u̯ei̯d- (independent of any meaning specification).

The full set of protocols for cognate determination is set out extensively in [^8^SM3].

Cognate determinations are accompanied by comprehensive reference to the extensive research literature on the history of the Indo-European languages, particularly major reference works such as *Lexikon der indogermanischen Verben* (LIV) and *Nomina im indogermanischen Lexikon* (NIL)^47,48^. Additionally, most relevant and up-to-date etymological handbooks were also consulted at the individual branch level, e.g. for Anatolian^57,58^, Tocharian^59^, Greek^60–62^, Armenian^63^, Albanian^49–51^, Indic^64–66^, Iranian^65,67,68^, Baltic^69–72^, Slavic^73,74^, Germanic^75–77^, Italic and Romance^78–81^, and Celtic^82–85^. Where appropriate and necessary, references to more antiquated reference works such as Julius Pokorny’s *Indogermanisches etymologisches Wörterbuch* (IEW)^86^ were also included.

### Loanwords

Although our optimisation criterion 5 did serve to significantly reduce their incidence in the IE-CoR dataset, there inevitably remain plenty of cases of horizontal transfer, i.e. loanwords from one language into another. While language is transmitted mostly vertically (through succeeding generations of speakers), horizontal transfers between languages are nonetheless also ubiquitous in language histories. Indeed this is another level on which IE-CoR introduces a significant and necessary departure^2^ from previous approaches. Hitherto, loanwords have typically (e.g. in IELex^24^, used in^5,6^) been encoded by means of just a boolean ‘loanword’ flag on the borrowed lexeme. The lexeme entry itself was handled in either of two ways, depending on multiple variables, including whether the source language and word happened to be also Indo-European, also covered in the dataset, and without a shift away from that reference meaning. Under these conditions, a loanword lexeme could be (but in^24^ was not always) retained in the cognate set of that source word (albeit flagged as a loan); otherwise it had its own separate cognate set status. These alternative handlings introduce inconsistency, however, and permitted phylogenetic analyses in which known horizontal transmission was nonetheless analysed as vertical (see^2^ on^5^). To overcome these methodological issues, IE-CoR handles all loanwords not by boolean flags on individual loanword lexemes but structurally, at a level equal to cognate sets, as necessary to embody the complexity of the relationship between cognacy and horizontal transmissions through time.

Cognate sets in IE-CoR are defined by vertical transmission from a common ancestor. So when a lexeme is borrowed from one language to another, this loan event is taken to break the vertical transmission of cognacy. However, once a lexeme has been borrowed into a language’s lexicon, it can thereafter be transmitted vertically again, as usual. Indeed, a new cognate set can arise out of it, defined by common origin in the lexeme borrowed in the original loan event. In the meaning fish, for example, Latin *piscis*, English *fish*, and Irish *iasc* all descend vertically from the Proto-Indo-European root *pisk-. IE-CoR therefore groups these lexemes (among others in many other languages) into a first cognate set defined by origin in *pisk- (220). The Latin word *piscis* was also *borrowed*, however, into an early stage of Brythonic Celtic, and this borrowed word was thereafter inherited into modern Brythonic languages. IE-CoR therefore groups these modern Brythonic lexemes into a second, ‘loan event’ cognate set, defined by descent from the *piscis* loanword into early Brythonic (4362). This is independent of the first set, because the loan event broke vertical transmission (although the specification of this loan-event cognate set does also include a field to cross-reference it to the original *pisk- cognate set, to which the Latin source word belongs). Potentially in a similar time-frame, the same Latin lexeme was also borrowed into the Albanian clade, but through a separate loan event. That defines a third cognate set (5293), which groups together the lexemes in several modern varieties of Albanian which are all inherited from the loan event of Latin *piscis* into Albanian; this set is also cross-referenced to the first.

Alternatively, the same lexeme may have been borrowed into multiple languages which, while related, had already diverged by the time of the loan event. Here the borrowed lexemes cannot be considered to belong to the same cognate set, as they have not *descended* from a common ancestral form. Often such cases are marked by complex chains of loan events that are difficult to recover. Nonetheless, these lexemes were all borrowed from the same source lexeme, and for more efficient data handling they are grouped together in IE-CoR into a special type of loanword cognate set, designated a ‘parallel loan’ set. In the meaning hunt, for example, the Persian word *šekār* (the source lexeme for IE-CoR cognate set 5596) was borrowed into numerous languages of Northern India after they had already diverged. Loanwords of this type, too, can thus be identified and handled in alternative ways when exporting IE-CoR data for phylogenetic analysis^8^.

## Dataset

IE-CoR is part of the Cross-Linguistic Linked Data project (CLLD: https://clld.org)^12^, coordinating linguistic datasets covering the languages of the world. It follows the Cross-Linguistic Data Format (CLDF)^13^, commonly used in linguistics and designed to handle version control and long-term archiving. The IE-CoR dataset is curated in a public GitHub repository https://github.com/lexibank/iecor. This uses the cldfbench toolchain^14^ in order to create the CLDF data for distribution.

The choice of repository ensures that the history of changes to the dataset is fully transparent. The state of the repository at any given release can be browsed using tag-specific URLs of the form: https://github.com/lexibank/iecor/releases/tag/v1.2. Changes between releases can be compared using URLs of the form: https://github.com/lexibank/iecor/compare/v1.0…v.1.2. Officially versioned releases are listed at: https://github.com/lexibank/iecor/releases.

Released versions are archived and made accessible for the long term on Zenodo. This provides permanent DOIs for citing each individual version, at https://zenodo.org/records/13304537, as well as a version-independent DOI that always resolves to the latest version https://zenodo.org/records/8089433. From there, Zenodo provides metadata from which to identify any earlier versions.

An analysis of the phylogeny of the Indo-European family based on version 1.0 (https://github.com/lexibank/iecor/tree/v1.0) of the IE-CoR data has been published as^8^. We now launch here the updated IE-CoR 1.2 https://github.com/lexibank/iecor/tree/v1.2. This version incorporates minor data corrections with respect to version 1.0, some of which were added in response to suggestions from colleagues and critique in an online review (https://starlingdb.org/Texts/Review_Heggarty2023.html), two fewer languages, removed to evaluate the coding of phonological adaptation and borrowing in these languages, and one new language. The latest release version of the IE-CoR dataset may be explored via a publicly accessible web application at https://iecor.clld.org.

The IE-CoR dataset is made freely available under a ‘CC BY 4.0’ licence, i.e. Creative Commons Attribution 4.0 International licence — see https://creativecommons.org/licenses/by/4.0/legalcode.

## Data Records

Here IE-CoR 1.2 contains data entries for 25731 lexemes, across 160 languages, arrayed into 4981 cognate sets, assembled by 89 language specialists, referencing 355 distinct sources. The data are stored in data tables accessible at https://zenodo.org/records/8089433^87^ as a package of files, mostly in .csv format. Figure 2 (in the Background and Summary section above) illustrates schematically the overall relational structure of the IE-CoR dataset, through the cross-references between fields in the different data tables.

Here we first describe the raw .csv data files in that download package. The contents of these data files (at least the main fields in each) can also be searched, filtered, and mapped in geographical space across the Indo-European family using the IE-CoR ‘dataset explorer’ web application at https://iecor.clld.org. Figure 3 provides an illustration of how the data in several of the data tables appear in the web application, for the example meaning FIRE, on the interactive page at https://iecor.clld.org/parameters/fire.

In the bullet points below to describe each .csv data table, we also refer briefly to the page-view in the web application that corresponds to that data table. In many cases that page-view also provides dataset statistics that reflect relationships to other .csv data tables.

Two data tables contain reference metadata on authorship and referencing.

- In the authors.csv file, each row corresponds to one of the 89 authors who contributed to IE-CoR data collection. Each row has fields for the author’s name and the url of their personal website. These data, and cross-references to the language(s) that each author worked on, can also be viewed at https://iecor.clld.org/contributors.
- In the sources.bib file, each entry is a bibliographical reference, in BibTeX format, of one of the 10925 citations in support of cognate and lexeme determinations in the IE-CoR 1.2 dataset. These references can also be explored, searched, filtered and downloaded (in a range of other reference management formats) on the IE-CoR web application at https://iecor.clld.org/sources. The 10925 citations in IE-CoR 1.2 refer to 355 distinct sources.

Data on the languages covered within IE-CoR are stored in two further tables.

- In the languages.csv file, each row represents one of the 160 IE-CoR languages, with fields for language metadata such as name strings, ISO 639-3 code and Glottocode, along with text fields to specify and justify which particular variety of that language was targeted in IE-CoR. Further fields encode where each language fits into IE-CoR’s reference cladistic structure for the Indo-European family, through cross-references to the clade structure fields in the clades.csv file (see next bullet point below). Other boolean fields encode whether a language is a present-day or historical variety, or a conservative written variety potentially less subject to the usual mechanisms of language change. The language’s geographical context is specified in fields for latitude and longitude, along with a text field to justify why that particular location is taken as representative for that language. For each non-modern language, its chronological context is also specified, by fields that define its IE-CoR time-calibration, in most cases expressed as a distribution around a central reference date, expressed in years before 2000 CE (taken as effectively the ‘present’). A contributor(s) field identifies which IE-CoR author(s) worked on each language (corresponding to the rows in the authors.csv table, see above). The languages included in IE-CoR 1.2 can also be explored on the web application at https://iecor.clld.org/languages.
- The clades.csv file sets out the uncontroversial main clades of the Indo-European family. This is not intended to inform constraints in cladistic analyses, and was not used in^8^, but serves in IE-CoR only to facilitate practical purposes of data display, filtering and analysis. Each row represents a clade at some level, with numerical fields to allow for resolution of up to four nested levels of clade structure, and fields for the corresponding colour codes as used in the IE-CoR web application. The reference clade structure can also be explored at https://iecor.clld.org/clades.

Figure 1 (see Background & Summary section) maps all languages in the IE-CoR sample of the Indo-European family. Table 1, below, displays a selection of these languages, ancient and modern, together with the data in their clade affiliation fields, and basic statistics on data coverage for that language, e.g. statistics on how many of the 170 IE-CoR reference meanings could be covered in each of the partly attested historical languages.

**Table 1:** An illustration of data on languages, as stored in a selection of fields in the languages.csv data table, including date calibrations, for a selection of IE-CoR languages.

Language ID	Sort order	IE-CoR Language Name	Closest Glottocode	Closest ISO 639-3	Specification of which variety of this language is targeted in IE-CoR	Author ID	Latitude	Longitude	Non-modern	Date Calibration Fields					
										Distrib’n type	Normal		Log-normal		
ID	sort_order	Name	Glottocode	ISO 639-3 code	Variety; Description	Author_ID	Latitude	Longitude	historical	distribution	normal Mean	normal StDv	log Normal Mean	log Normal Offset	log Normal StDv
80	1	Hittite	hitt1242	hit		26;4	40.01	34.62	TRUE	Normal	3450	125			
82	4	Tocharian B	tokh1243	txb	Archaic/early Tocharian B of the Kuča region, atte…	24	41.72	82.96	TRUE	Normal	1350	75			
110	6	Greek: Ancient	anci1242	grc	Classical Attic. For phonological description and…	4	37.96	23.73	TRUE	Normal	2400	32			
129	15	Armenian: Classical	clas1249	xcl		6	40.17	44.29	TRUE	Normal	1550	25			
143	19	Albanian: Standard	alba1267	sqi	Standard Albanian — see also the location justific…	9	41.11	20.08	FALSE						
105	21	Vedic: Early	vedi1234	san		25	34.04	72.36	TRUE	Offset log -normal			700	3000	0.8
10	25	Bengali	beng1280	ben		44	22.57	88.36	FALSE						
128	46	Avestan: Younger	aves1237	ave		69	31.54	53.63	TRUE	Offset log -normal			350	2550	0.8
271	48	Sogdian	sogd1245	sog		45	39.7	66.98	TRUE	Normal	1150	25			
46	76	Lithuanian	lith1251	lit	Standard Lithuanian	10	54.84	23.17	FALSE						
100	78	Old Church Slavonic	chur1257	chu	Old Church Slavonic sensu stricto, as documented…	11	40.65	22.9	TRUE	Normal	1000	50			
55	88	Polish	poli1260	pol	Standard Polish	11	52.23	21.01	FALSE						
302	102	Old Icelandic	oldn1244	non		73	64.26	−21.12	TRUE	Normal	800	15			
22	112	English	stan1293	eng	British English, with lexeme d…	20;2	51.75	−1.26	FALSE						
295	119	Old High German	oldh1241	goh	East Franconian, the dialect o…	73	50.55	9.67	TRUE	Normal	1170	5			
112	124	Latin	lati1261	lat	Classical Latin (literary standard of the Roman La…	4	41.89	12.48	TRUE	Normal	2050	75			
25	136	French	stan1290	fra	Standard, with lexical usage o…	2	47.24	0.69	FALSE						
239	150	Middle Welsh	midd1363	wlm		32	52.2	−3.6	TRUE	Normal	650	75			
127	158	Old Irish	oldi1245	sga	Primarily based on Old Irish glosses	1	54.35	−6.66	TRUE	Normal	1250	50			

An illustration of data on languages, as stored in the languages.csv data table, including date calibrations and selected statistics, for a selection of IE-CoR languages.

The meanings and lexemes covered in IE-CoR are stored in two further data tables.

- In the parameters.csv file, each row represents one of the 170 IE-CoR reference meanings (i.e. IE-CoR’s “semantic parameters”). For each meaning there is a text field for the meaning identifier (using English as the meta-language), and further fields to specify more narrowly and unambiguously the particular sense targeted in IE-CoR, in order to maximise consistency in lexeme determination across all languages. These fields include a brief text description and an optional disambiguation field for cases where the English word is ambiguous (e.g. bark is the noun meaning the outer layer of a tree, not the verb (or noun) for the sound made by a dog). Another field sets out a single illustrative context sentence: each lexeme entry should be the default term used in that language in this illustrative context. There is also an extensive markdown field for the full IE-CoR definition of this meaning, and the specific sense targeted in IE-CoR. Other fields cross-reference each meaning to the most closely corresponding concept identifier in the Concepticon catalogue of concept sets, and link directly to that entry in https://concepticon.clld.org^40^. The IE-CoR web application also displays basic dataset statistics for each meaning: how widely covered that meaning is across the IE-CoR language sample (since certain meanings may not be attested in the limited corpora for some historical languages); how many independent cognate sets are found in this meaning across the IE-CoR languages; and how many of those cognate sets originated in loan events. These statistics can be explored at https://iecor.clld.org/parameters, and are shown for an illustrative sample of IE-CoR meanings in Table 2.

**Table 2:** An illustration of data on meanings, as stored in a selection of fields in the parameters.csv data table, for a selection of IE-CoR reference meanings.

IE-CoR Meaning ID	IE-CoR Meaning Name	IE-CoR Illustrative Context	IE-CoR Meaning Definition (first 100 characters)	Nearest equivalent in Concepticon	
ID	Name	Description_md		Concepticon_ ID	Concepticon_Gloss
14	black	Charcoal is **black**.	The most basic colour term (usually adjectival) seen as the natural antonym of [white](../wiki/Meani…	163	BLACK
23	cold	The rocks get hot during the day and **cold** at night.	The default antonym of [hot](../wiki/Meaning:-hot). The most basic term, typically adjectival, that…	1287	COLD
30	dirty	This cup is clean and that one is **dirty**.	The most generic term, typically adjectival, as the antonym of _clean_.The target is the literal…	1230	DIRTY
40	eye	She closed one **eye**.	The basic term for the human eye. Follow normal usage in the language, rather than technical anatomi…	1248	EYE
50	fire	He was cold, so he moved closer to the **fire**.	Most generic, basic and default noun for _fire_, preferably applicable both to the concept of fire i…	221	FIRE
51	fish	I can see a **fish** swimming in the water.	The most generic noun for a live fish.Avoid terms specific to caught, cooked or prepared fish, e….	227	FISH
58	foot	She has injured her **foot** so she can’t walk.	The most generic noun for the foot as a part of the human body. In many languages the basic term m…	1301	FOOT
60	freeze	When water **freezes**, it becomes ice.	The basic term, normally an intransitive verb, for water turning to ice. The same term is in many la…	1431	FREEZE
186	hot	The rocks get **hot** during the day and cold at night.	The default antonym of [cold](../wiki/Meaning:-cold). The most basic term, typically adjectival, th…	1286	HOT
80	hunt	He is **hunting** animals in the forest.	The most generic transitive verb for hunt, in the prototypical sense of a human tracking or chasing…	1435	HUNT
88	know	They’re coming tomorrow.” — “Yes, I **know**.”	The most generic verb for knowing **a fact**, having information about something. Beware of the pol…	1410	KNOW (SOMETHING)
89	lake	Her house is beside the **lake**.	The most generic noun for a naturally occurring body of still (and normally, fresh) water in the lan…	624	LAKE
102	moon	I saw the **moon** and the sun in the sky.	The term selected should be the basic, default word for the (earth’s) moon.In most languages thi…	1313	MOON
122	red	Blood is **red**.	The most basic colour term (usually adjectival), typically of some internal human body parts (e.g. t…	156	RED
137	see	I **see** him every day on the road.	The most generic transitive verb for see, in the literal, prototypical sense of perceiving with the…	1409	SEE
151	snake	He jumped when he saw the **snake**.	The most generic term for a snake as a general body-form of animal, in popular perception seen proto…	730	SNAKE
188	water	She was thirsty, so she drank some **water**.	The most generic, basic and default noun for _water_. In many languages this will be the same term a…	948	WATER

An illustration of data on meanings, as stored in the parameters.csv data table, for a selection of IE-CoR reference meanings.
- In the forms.csv file, each row represents a lexeme that in a given language was determined as the primary, single lexeme best fitting for a given IE-CoR meaning specification. Each row includes an IE-CoR lexeme identifier number, and four Unicode text fields for various representations of this lexeme. For immediate comparison, all lexemes are given a representation in Roman script. In languages normally written in other scripts, this Roman script field therefore contains a standard transliteration, while the native orthography is entered in a dedicated separate field, using the Unicode characters for that script. Both fields follow the established citation conventions used in dictionary and grammar resources for that language. There are also fields for both phonemic and phonetic transcriptions (where provided by the coder), both using the Unicode character set for the International Phonetic Alphabet (IPA). In addition, there are two further separate fields for transcriptions segmented and standardised to the BIPA conventions of CLTS^88,89^, and compatible with the lingpy Python library for quantitative tasks in historical linguistics: https://lingpy.org and https://github.com/lingpy^90,91^. Further fields include: a gloss, i.e. established English translation(s) of this lexeme; any additional notes on the lexeme, particularly as relevant to justifying why it fits the targeted IE-CoR meaning specification better than potential near-synonym lexemes in the same language; and hyperlinks direct to online dictionary resources, as featured also on the corresponding pages on the web application at https://iecor.clld.org/values. IE-CoR 1.2 contains 25731 lexemes (i.e. rows in the forms.csv file), of which 18801 include phonetic transcriptions and 20313 phonemic transcriptions. A sample data record is shown in Table 3.

**Table 3:** An illustration of data on lexemes, as stored in a selection of fields in the forms.csv data table, for a selection of IE-CoR reference meanings, in Ancient Greek as an example language.

IE-CoR Lexeme ID	Language ID	Meaning ID	Roman(ised)	In native script	PhoneMic transcription	PhoneMic transcription segmented	PhoneTic transcription	PhoneTic transcription segmented	Comments (first c. 40 characters)	External dictionary reference^*^
ID	Language_ID	Parameter_ID	Form	native_script	Phonemic	Phonemic_Segments	phon_form	Segments	Comment	url
110-14-1	110	14	mélas	μέλας	mé̞laːs	m é̞ l aː s	mé̞laːs	m é̞ l aː s	G.sg. μέλανος; μέλᾱς, μέλαινα, μέλᾰν	me/las
110-23-1	110	23	psychrós	ψυχρός	psyːkʰró̞s	p s yː kʰ r ó̞ s	psyːkʰró̞s	p s yː kʰ r ó̞ s	G.sg. ψυχροῦ; ψυχρός, ψυχρά, ψυχρον.	yuxro/s
110-30-1	110	30	rhyparós	ῥυπαρός	hryparó̞s	h r y p a r ó̞ s	hryparó̞s	h r y p a r ó̞ s	G.sg. ῥυπαροῦ; ῥυπαρός, ῥυπαρά, […].	r(uparo/s
110-40-1	110	40	ophthalmós	ὀφθαλμός	o̞pʰtʰalmó̞s	o̞ pʰ tʰ a l m ó̞ s	o̞pʰtʰalmó̞s	o̞ pʰ tʰ a l m ó̞ s	G.sg. ὀφθαλμοῦ.	o)fqalmo/s
110-45-1	110	45	phobéomai	φοβέομαι	pʰo̞bé̞o̞mai̯	pʰ o̞ b é̞ o̞ m a i̯	pʰo̞bûmai̯	pʰ o̞ b û m a i̯	Inf. φοβεῖσθαι.	fobe/w
110-50-1	110	50	pỹr	πῦρ	pŷːr	p ŷː r	pŷːr	p ŷː r	G.sg. πῠρός	pu = r
110-51-1	110	51	ichthỹs	ἰχθῦς	ikʰtʰŷːs	i kʰ tʰ ŷː s	ikʰtʰŷːs	i kʰ tʰ ŷː s	G.sg. ἰχθῡ́ος.	i)xqu = s
110-58-1	110	58	poús	πούς	púːs	p úː s	púːs	p úː s	G.sg. ποδός.	pou/s
110-60-1	110	60	pḗgnumai	πήγνυμαι	pɛ́ːɡnumai̯	p ɛ́ː ɡ n u m a i̯	pɛ́ːɡnumai̯	p ɛ́ː ɡ n u m a i̯	Inf. πήγνυσθαι; intrans.	ph/gnumi
110-186-1	110	186	thermós	θερμός	tʰe̞rmó̞s	tʰ e̞ r m ó̞ s	tʰe̞rmó̞s	tʰ e̞ r m ó̞ s	G.sg. θερμοῦ; θερμός, θερμή, θερμόν.	qermo/s1
110-80-1	110	80	thēreúō	θηρεύω	tʰɛːréu̯o̞ː	tʰ ɛː r é u̯ o̞ː	tʰɛːréu̯o̞ː	tʰ ɛː r é u̯ o̞ː	Inf. θηρεύειν. Cf. θήρ ‘wild animal’ […].	qhreu/w
110-88-1	110	88	oĩda	οἶδα	óì̯da	ó ì̯ d a	óì̯da	ó ì̯ d a	Inf. εἰδέναι. Irregular verb; cf. notes in […].	oi) = da
110-89-1	110	89	límnē	λίμνη	límnɛː	l í m n ɛː	límnɛː	l í m n ɛː	G.sg. λίμνης	li/mnh
110-102-1	110	102	selḗnē	σελήνη	selɛ́ːnɛː	s e l ɛ́ː n ɛː	selɛ́ːnɛː	s e l ɛ́ː n ɛː	G.sg. σελήνης	selh/nh
110-122-1	110	122	erythrós	ἐρυθρός	e̞rytʰró̞s	e̞ r y tʰ r ó̞ s	e̞rytʰró̞s	e̞ r y tʰ r ó̞ s	G.sg. ἐρυθροῦ; ἐρυθρός, ἐρυθρά, […].	e)ruqro/s
110-137-1	110	137	horáō	ὁράω	ho̞ráo̞ː	h o̞ r á o̞ː	ho̞rô̞ː	h o̞ r ô̞ː	Inf. ὁρᾶν. Has suppletive aorist εἶδον […]	o(ra/w
110-151-1	110	151	óphis	ὄφις	ó̞pʰis	ó̞ pʰ i s	ó̞pʰis	ó̞ pʰ i s	G.sg. ὄφεως	o)/fis
110-188-1	110	188	hýdōr	ὕδωρ	hýdo̞ːr	h ý d o̞ː r	hýdo̞ːr	h ý d o̞ː r	G.sg. ὕδατος	u(/dwr

*The external dictionary lookup text is appended to the root path specified by language, in this case: http://www.perseus.tufts.edu/hopper/text?doc=Perseus:text:1999.04.0057:entry=.

An illustration of data on lexemes, as stored in the forms.csv data table, for a selection of IE-CoR reference meanings, using Ancient Greek as an example language.

Two further data tables store the IE-CoR data on cognate sets, and a third table relates every lexeme entry in forms.csv to the cognate set it belongs to.

- In the cognatesets.csv file, each row represents one of the cognate sets into which lexemes for the same IE-CoR meaning in different languages are grouped by shared origin. As well as its IE-CoR ID number, each cognate set is identified by an IE-CoR reference form (using Unicode text in Roman script, plus IPA characters where needed), in one of the IE-CoR reference languages. In most cases this means a reconstructed ancestral form, either in Proto-Indo-European or in a more recent proto-language ancestral to a major intermediate clade, such as Proto-Slavic. Alternatively, IE-CoR reference forms can be attested written forms in a very well documented ancient language judged to be close to the ancestor of a clade, e.g. literary Classical Latin, rather than reconstructed Proto-Romance. Additional boolean fields record whether the cognate set is judged to be ideophonic or derived in parallel. There are also text fields for notes and for justifications of cognate set determination, as supported by bibliography fields for extensive references to discussions of that specific cognate set in the rich literature on Indo-European linguistics. The cognatesets.csv file can also accommodate alternative viewpoints. One field allows one cognate set to be cross-referenced to another, in cases where a hypothesis has been put forward that both in fact derive from the same source. A second field encodes a determination of the degree of support for that hypothesis in the relevant literature. IE-CoR thus allows dataset exports at several different levels, whether assuming a conservative consensus position or specific hypotheses on Indo-European reconstruction.
  A further dedicated field is provided in order to cross-reference a cognate set to any equivalent entry used also in other meanings, to thus link them as a common cognate superset (see above under Methods).
  All of the 4981 cognate sets in IE-CoR can be explored on the web application at https://iecor.clld.org/cognatesets. A sample cognate set record is provided in Table 4.

**Table 4:** An illustration of data on cognate sets, as stored in a selection of fields in the cognatesets.csv data table, for the example meaning FIRE.

IE-CoR Cognate Set ID	IE-CoR Cognate Set Root Reference Form	IE-CoR Root Reference Language	Cognacy justification (first 50 characters)	Bibliographical references (first 50 characters)	Proposals as cognate to other IE-CoR cognate set	Cognate cross-meaning ‘Superset’ ID
ID	Root_Form	Root_Language	Justification	Source	proposedAsCognateTo_pk	supersetid
219	*péh₂-u̯r̥, *ph₂-u̯én-	Proto-Indo-European	Anatolian, Tocharian, Hellenic, Armenian, Baltic,…	66[1260-1261{S.v. πῦρ, πυρός ‘fire’ < PIE neuter h…		
396	*gʷʰer-	Proto-Indo-European	Albanian lexemes generally derived from PIE *gʷʰer…	105[525{S.v. Alb. zjarr ‘fire’ < PIE *gʷʰer-es- (c…		95
774	*h₁n̥gni-	Proto-Indo-European	Indic, Balto-Slavic, and Italic lexemes, generally…	172[I:44-45{S.v. Ved. agní- ‘Feuer| das vergöttlic…	6119	
877	focus	Latin	Romance lexemes from Latin focus ‘hearth, fireplac…	54[228-229{S.v. Lat. focus ‘hearth, fireplace’. Th…		
1001	*tep-	Proto-Indo-European	Celtic lexemes continuing derivatives of PIE *tep-…	50[375{S.v. Proto-Celtic *tefnet- ‘fire’ derived f…		290
1314	*ailida-	Proto-Germanic	Etymology uncertain but generally connected to PIE…	165[11{S.v. Proto-Germanic *ailida- ‘fire’, a form…	6529	
1482	*bʰeh₂-	Proto-Indo-European	Modern Greek lexemes derived from Ancient Greek φῶ…	66[1551-1552{S.v. φάος ‘light, daylight’ < PIE *bʰ…		29
1957	*h₂eh₁-	Proto-Indo-European	Lexemes continuing Proto-Iranic *ātr-, *ātar-, a d…	141[257{S.v. *h₂eh₁- ‘heiß sein’ (not in IEW). The…		123
2502	*branda-	Proto-Germanic	IE root etymology unclear, cf. [LIV²](src-141) 92-…	397[146{Cf. s.v. NHG Brand (MHG brant, OHG brant,…		
3629	raxnig	Wakhi	Presumably an early loanword from Persian rušnāyī…	321[304{S.v. Wa.rəxnig ‘огонь, пламя‘, apparently…		
4472	gindara	Sinhalese	Loanword from Pāli aggini-, gini- ‘fire’, replaci…	442[3{Cf. s.v. 55 55 agní- m. ‘fire’. RV. > OSi. a…		
5016	*dʰegʷʰ-	Proto-Indo-European	Derived from PIE *dʰegʷʰ- ‘to burn’ ([Bailey 1979]…	320[164{S.v. Khot. dai (1) ‘fire| caustic stuff’….		71
5348	krak	Armenian: Classical	Modern Armenian lexemes from Classical Armenian kr…	79[378{S.v. Arm. krak ‘fire’, some proposed Indo-E…		
5557	vatra	Serbo-Croat	A Wanderwort of obscure origin, cf. [Georgiev et a…	598[1: 279{Some scholars assume here a native word…		
5869	λούτσ̌ι / loútši	Greek: Italiot	Loanword from dialectal southern Italian ([Rohlfs…	587[301-302{S.v. Italiot *λούκιον ‘Feuer’, loanwor…		
6119	*Hengl-	Proto-Indo-European	Indo-Iranic lexemes from Vedic áṅgāra- ‘glowing ch…	172[I:48{Cf. s.v. Ved. áṅgāra- ‘Kohle’, probably n…	774	
6157	ἑστία / hestía	Greek: Ancient	From Ancient Greek ἑστία ‘hearth, fireplace, altar…	452[600{From Ancient Greek ἑστία ‘hearth, fireplac…		
6159	*h₂ep-	Proto-Indo-European	Pontic Greek άψιμο(ν) a derivative of the aorist s…	464[{άψιμο < aor. stem AG ἅψ- (of ἅπτω ‘kindle’, c…		
6160	ἐσχάρα / eschára	Greek: Ancient	Tsakonian (ι)κ̔άρα < Ancient Greek ἐσχάρα ‘hearth’…	66[472{S.v. AGk. ἐσχάρα ‘hearth, house, sacrificin…		
9763	*dei̯-	Proto-Indo-European	Assamese jui ‘fire’, continuing Vedic jyótis- ‘lig…	442[292{S.v. 5300 jyṓtis n. ‘light, moonlight’ RV….		46

An illustration of data on cognate sets, as stored in the cognatesets.csv data table, for the example meaning FIRE.
- The loans.csv file stores additional data only on the subset of cognate sets that derive from a loan event. Each row represents one of these loan-event cognate sets, with its ID field cross-referenced to the corresponding entry within the cognatesets.csv data table. Additional fields identify the source language from which this loan was taken, and the ID of the cognate set to which that source lexeme belongs, if also present in the IE-CoR dataset. A text field is available for notes on this loan-event cognate set, while a boolean field is used to designate sets that do not derive from a single loan event, but are cases where the same source lexeme was borrowed independently, in parallel, into multiple other languages.
- Cognate sets of the loan-event type can be explored on the web application at https://iecor.clld.org/cognatesets, where the fields from the loans.csv file appear in additional columns for loan-event data. On the web application, to filter to cognate sets only of the loan-event type use the URL https://iecor.clld.org/cognatesets?sSearch_6=True. Alternatively, to filter to cognate sets only of the *parallel* loan subtype, use the URL https://iecor.clld.org/cognatesets?sSearch_7=True.
- Every lexeme is associated to its corresponding cognate set via the cognates.csv file, which is a simple relational table linking lexeme identifiers from the forms.csv file to cognate set identifiers in the cognatesets.csv file.

As well as the raw data files just described, much of the same data can be visualised and explored using the IE-CoR web application at https://iecor.clld.org (see Usage Notes).

## Technical Validation

The specific nature of language data determines which particular forms of technical validation are necessary, most appropriate, or possible at all. As noted above in the Background & Summary section, most language data are non-discrete, not measurable instrumentally, and meaningful only in the context of a wider system. Languages also differ widely in how they organise the relationships between the sound signal, word forms, and meanings. The same technical measures (e.g. sound frequencies) are not equally significant in all languages; some degree of abstraction in analysis is inherently necessary.

Data were coded only by specialist analysts in each language or clade, hence the 89 authors in the IE-CoR consortium (whereas most previous datasets were coded by just one or a few linguists^2^). Previous cognate datasets for Indo-European have been undermined above all by serious dataset inconsistencies^2^, however, so it was especially crucial to validate IE-CoR data for consistency. In general, IE-CoR implemented strict protocols on many levels to minimise inconsistency (see under the Methods section above).

Specialists in the same languages cross-validated each other’s lexeme and cognacy determinations, to come to a confirmed consensus coding. This validation was applied especially to historical languages with limited attested corpora, and to known problematic cases in modern languages (e.g. lexeme determinations for words for throw in languages of the Romance clade, or hot in Germanic).

Another IE-CoR validation requirement was for extensive cross-referencing of cognate determinations to the corresponding reconstructions in standard reference works in Indo-European linguistics^47,48^. For lexeme determinations, meanwhile, individual hyperlinks are provided to the corresponding entries in authoritative reference works such as the *Oxford English Dictionary* or the dictionary of the *Real Academia Española*. Their etymologies also stand as further validation of IE-CoR cognacy determinations. Lexeme entries in the IE-CoR web application link directly to these validation sources.

Text transcriptions were validated against established technical standards and conventions. In particular, segmented versions of the phonemic and phonetic transcription fields were produced and are included in the forms.csv file in the dataset. These tolerate only characters and combinations permissible in the International Phonetic Alphabet, and only in the corresponding Unicode characters. All transcriptions were validated computationally against the BIPA transcription system of the CLTS dataset^88,89^, and identified errors were then corrected. Representations of lexemes in Romanised script were validated against established transliteration standards for conversion from other native scripts (e.g. Cyrillic for Russian). Given the number of contributors, not all of whom are specialists in phonology, and given that cognacy, not phonology, is the primary purpose and focus of this dataset, further research to attempt to reconcile different standard transcription practices across languages remains a task for a future version of IE-CoR, to fully optimise it for phonological comparison.

Particularly important was validation for consistency across the dataset as a whole. To this end, IE-CoR applied strict overall constraints. Multiple, near-synonymous lexeme entries for the same meaning were rejected if they exceeded the maximum tolerance of 4% in any one language, and final codings were made by cross-validation across multiple linguist coders. Inconsistent over-sampling of language varieties was avoided by imposing a minimum threshold of 4% difference in cognacy. Dataset consistency was also validated by tools and statistics developed for rigorous, fine-grained cross-checking of data tables and exports from them (e.g. in nexus format). A range of dataset statistics were constantly reviewed, as error-checking failsafes for data consistency and completeness. One specific validation, for example, was implemented to ensure that no lexeme was left unmapped to any cognate set, i.e. without an explicit cognate determination. Another validation test detected any cases where a single lexeme was mapped to multiple cognate sets, only permitted in tightly defined and highly restricted circumstances, and therefore to be individually verified. Any lacunae or inconsistencies detected by these statistical controls were corrected as they were found. (Previous datasets suffered from script bugs that left inconsistencies in data exports, unnoticed in past phylogenetic analyses of such data^2^) Many of these statistics can be viewed on the pages of the IE-CoR web application. IE-CoR validation policy also developed tools to regularly verify triangular distance matrices of shared cognacy between all pairs of languages, to confirm conformity with expectations from expert qualitative assessments of relative language similarity.

Further validations are possible against external, historical knowledge. In previous Indo-European cognate datasets, dataset inconsistency led to obvious artefacts in results. Phylogenetic analyses returned excessively long (or short) branches for languages whose development is in fact well documented during the historical period, e.g. from Latin to the modern Romance languages, from Ancient to Modern Greek, or the divergence of (West) Norse since the settlement of Iceland and the Faroes^2^. In comparable phylogenetic analyses of the IE-CoR dataset, the artefactual branch lengths disappear, and split date estimates correspond much more closely to known divergence histories^8^. Similar external validations were possible for the two IE-CoR language date calibrations that are uncertain (because no original texts survive). Even when these calibrations for Early Vedic and Younger Avestan were simply removed, the analysis returned date estimates very closely in line with those assigned to them in the IE-CoR languages table^8^. More generally, results from previous datasets were highly sensitive to changed modelling assumptions^2^, whereas results from IE-CoR data prove much more stable, a further form of validation of the greater consistency of the IE-CoR dataset^8^.

## Usage Notes

The entire IE-CoR dataset can be explored through the web application at https://iecor.clld.org, and is free to download from https://zenodo.org/records/8089433^87^. The requirements.txt file at https://github.com/lexibank/iecor/tree/master/cldf lists all Python > = 3.7 packages used in building the data files, while the cldf-metadata.json file sets out the dataset structure and relationships between the linked fields in all .csv data-tables, as detailed above in the Data Records section.

The web application allows data to be visualised as a customisable map of the main Indo-European language area, and the data tables can be searched and filtered. As well as the data from the corresponding base table, several of the page-views additionally show important dataset statistics, calculated not just from that single table itself, but from cross-references within it to the corresponding entries in other linked data tables, particularly the cognates.csv file. The meanings page at https://iecor.clld.org/parameters, for example, includes statistics on:

- how widely covered each meaning is across the IE-CoR language sample (since certain meanings may not be attested in the limited corpora for some historical languages);
- how many independent cognate sets are found in each meaning across the IE-CoR languages;
- how many of those cognate sets originated in loan events.

Each page-view also allows the data table displayed to be downloaded individually, including any cross-table statistics, and in a range of file formats.

## Data Availability

The IE-CoR data-set and web application do not use any custom code. The web application is based on the Cross-Linguistic Linked Data (CLLD) Framework, see https://clld.org. The entire Python >  = 3.8 script code and parameters, for generating both the dataset and the web application, are freely accessible. The dataset is at https://zenodo.org/records/8089433 and the web application at https://github.com/clld/cobl2.
